# Insights in nonlinear ground response in volcanic environments from distributed dynamic strain sensing

**DOI:** 10.1038/s41598-025-20368-0

**Published:** 2025-09-24

**Authors:** Sergio Diaz-Meza, Philippe Jousset, Gilda Currenti, Lucile Costes, Charlotte M. Krawczyk

**Affiliations:** 1 GFZ Helmholtz Centre for Geosciences, Potsdam, 14473 Germany; 2 Institute of Applied Geosciences, Technical University of Berlin, Berlin, 10587 Germany; 3 Istituto Nazionale di Geofisica e Vulcanologia (INGV), Catania, 95125 Italy; 4 Institut des Sciences de la Terre, Grenoble, 38610 France

**Keywords:** Distributed acoustic sensing, Site response, Nonlinearity, Distributed dynamic strain sensing, Etna, Volcanoes, Geophysics, Seismology, Volcanology, Natural hazards, Civil engineering

## Abstract

Volcanic environments are often characterized by frequent explosive activity and complex ground features. Explosions can couple into the ground, triggering ground response (GR) influenced by near-surface properties. While GR resulting from seismic input is well-studied, GR generated by air-to-ground coupling of volcanic explosions remains poorly understood. Investigating this phenomenon is crucial for understanding near-surface material dynamics and improving volcanic hazard assessments. To study explosion-induced GR, a multi-parametric network was deployed near Mt. Etna’s summit craters in 2019, where GR had been previously observed. The network includes broadband seismometers, infrasound sensors, and a fibre optic cable for distributed dynamic strain sensing (DDSS). Over 65,000 explosions were recorded, with some triggering high-frequency GR signals (10–50 Hz) in the DDSS data. These high-frequency signals, embedded in low-frequency explosions (0.7–4 Hz), amplify upon coupling into the ground. We also classified the explosions using waveform similarity, and GR signals were analysed using an adapted approach incorporating temporal and spatial dimensions. Strain rate vs. pressure rate relationships derived from classified signals were interpreted in terms of either linear elastic or hyperelastic near-surface behaviour. Despite no clear consensus towards which mechanical model describes best the ground behaviour, we suggest a nonlinear site amplification driven by mechanical particle interactions rather than near-surface layer resonance.

## Introduction

Ground response (GR) refers to the behaviour of a local site under applied stress, including the amplification and damping of wavefield components under linear elastic (usually referred as site response^1,2^) and nonlinear elastic conditions^3^. Such behaviour is influenced by the ground composition^4^ and environmental factors such as moisture and temperature^5^.

Site response is understood as the amplification and/or damping of certain seismic wavefield components (frequencies) passing through a subsurface structure such as dykes, geological and stratified layers^6,7^. In linear elasticity, the amplification of wavefield components can be a consequence of resonance effects. For a resonance to occur, the frequency of the seismic wave that interacts with the ground layer must match the resonance frequency of the ground layer. In other words, the wavelength of the seismic wave must be equal to the thickness of the ground layer^8^. In case of the near-surface resonance effects (ground motion amplification), a quarter of the wavelength must match the thickness of the near-surface layer^9–11^. Higher harmonics from the fundamental resonance frequency can also be present and amplified^12^. In addition, during resonance, the multiple wave reflections of the amplified frequencies within the ground layer create the effect of stationary waves, which induce motion amplification^8^.

On the other hand, under nonlinear elastic conditions the response is more complex. Here, changes in the proportion between direct load from seismic waves and the resulting deformation on the ground occur, meaning that such a proportion is not constant and cannot be described linearly^3^. For example, non-consolidated soils can exhibit hyperelastic behaviour^3,6^. Hyperelasticity describes multiple stages of softening (high strain rates under small stress rates) and stiffening (low strain rates under high stress rates) as applied stress increases, normally seen in laboratory under uniaxial compression experiments^3^. Under nonlinearity, the site response depends mainly on the distribution and composition of the ground^4^, as they dictate the current elastic state of the site under a specific load. Therefore, the subsurface acts as a nonlinear filter and affects the wavefield.

Seismic waves are not the only triggers of nonlinear ground responses^6^. Blasts, such as explosions, and strong acoustic waves in the air can also interact with near-surface layers, causing notable ground responses^13,14^. These strong acoustic waves can damage nearby infrastructure and undermine the stability of foundation where infrastructures lie upon^13,14^, making it essential to understand their impact.

Active volcanic environments, characterized by frequent explosion events and complex geological structures, provide ideal natural settings for studying ground response behaviours triggered by acoustic waves. To investigate the nonlinear ground response from explosions further, we selected Mt. Etna, Europe’s largest volcano. Mt. Etna is notable for its continuous volcanic activity and for generating seismo-acoustic signals across a broad frequency range (0.05–100 Hz)^15–17^. This volcano also exhibits complex subsurface features, where unconsolidated deposits (e.g., scoria) and structural elements, such as dyke intrusions, lava flows, and faults, are prevalent^15,18,19^.

Possible nonlinear ground responses due to volcanic explosions at Mt. Etna have been observed in a previous study^15^. The study recorded differences between the frequency components of explosion signals captured by an infrasound sensor at the surface and by subsurface ground sensors (15 cm deep). The work identified these frequency discrepancies as nonlinear ground responses due to the resonance effect of the loose scoria layer (near-surface). However, it also noted that not all volcanic explosions produce detectable high-frequency ground signals, indicating that the mechanics of this response remain incompletely understood. Here we clarify these mechanisms by characterizing and analysing multi-parameter data from Mt. Etna, identifying key factors influencing nonlinear ground responses and improve predictive models for improving hazard assessments.

**Fig. 1:**
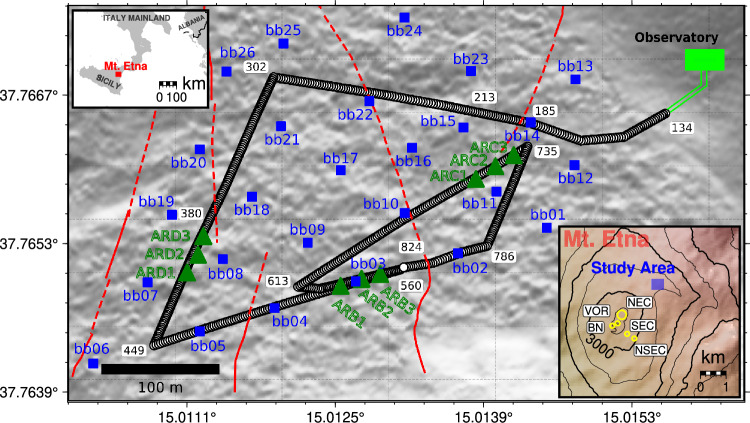
Map of the study area (Piano delle Concazze; PDC, blue area in Mt. Etna map), located  2.1 km NE from the five main craters of Etna (North-East Crater; NEC, Voragine; VOR, Bocca Nova; BN, South-East Crater; SEC, and New South-East Crater; NSEC). The deployed multi-parametric network is comprised by: 1) one 1.5 km long fibre optic cable (black circled line) interrogated from the observatory (green square), with some channels indices indicated by black numbers in white areas, 2) 26 broadband (BB) seismometers (blue squares) and 3) 9 infrasound sensors (green triangles). Confirmed and inferred fault in the area are shown as continuous and dashed red lines, respectively. Basemap done with QGIS 3.28 (https://qgis.org/), and pyGMT package^45,46^.

To better understand nonlinear ground responses, we deployed a multi-parametric instrument network at Mt. Etna to record and analyse ground responses to volcanic explosions over three months (Jul - Sep) in 2019 (Fig. 1; see Methods: Data acquisition). The study area, Piano delle Concazze (PDC), is located at around 2.1 km NE from the active craters at the summit of Mt. Etna^15^. It also spans  0.24 $\hbox {km}^{2}$ and features loose scoria deposits overlying fracture zones, where nonlinear responses were previously observed^15^. Our network consists of 26 broadband (BB) seismometers, 9 infrasound sensors, and a 1.5 km fibre optic cable for Distributed Dynamic Strain Sensing (DDSS), also known as Distributed Acoustic Sensing (DAS).

We analyse the high frequency (10 - 50 Hz) ground response (GR) associated to the air-to-ground coupling of acoustic waves produced by more than 8000 volcanic explosions. We inspect and classify volcanic explosions from infrasound data and the associated GR in DDSS data. Since our measurements come from waves travelling through the medium, the loading is dynamic (cyclic waves) instead of static. Therefore, to assess local elastic behaviours, we analyse the relation between strain rate and pressure rate peak-to-peak (p-p) amplitudes from classified GR events with classified explosion. Additionally we computed horizontal vs. vertical (H/V) component spectral analysis from BB network data to validate underlying response mechanisms and ground properties observed with DDSS.

## Results

### Volcanic explosions catalogue & classification

**Fig. 2:**
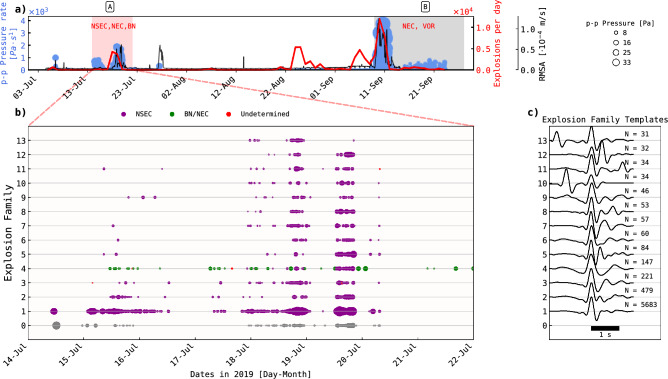
Detected volcanic explosions in infrasound. **a)** Explosions for the entire recording period (01-Jul until 24-Sep). The red line indicates the number of explosions per day. Blue dots represent each explosion, with their size indicating the maximum peak-to-peak (p-p) pressure amplitudes and their y-axis indicating their maximum p-p pressure rate amplitudes. The black curve is the hourly average root mean square (RMS) seismic amplitude of all three components for station bb15, filtered between 0.5 and 5 Hz. The red area (A) indicates an eruption period (14-Jul until 22-Jul). Only data from this period was processed in this study. The gray area (B) indicates an eruption period with the respective crater acronyms that were active^26^, not used in analysis since it may introduce signal artifacts in the GR signals. **b)** The first 13 families of volcanic explosions with highest number of members from the eruption period A. The color of the dots indicate the crater source according to back-azimuth estimations from infrasound array (see Methods: Detection of volcanic explosion). Red colored dots indicate explosions whose source could not be determined due to high back-azimuth error estimation. Explosions in gray represent non-classified waveforms. **c)** Examples of waveform templates obtained for some of the families reported in subfigure **b** (normalized amplitudes). The number of members per family are indicated at the right side of each.

During the three-month recording period (July 1 to September 24), we detected over 65,000 volcanic explosion over infrasound sensors (see Method: Detection of volcanic explosions) with common frequency content ranging from 0.7 to 4 Hz (Fig. 2a). We measured peak-to-peak (p-p) pressure and pressure rate for each event across all infrasound sensors, averaging the p-p values from all single infrasound sensors to obtain a single p-p value per acoustic event. p-p amplitudes between sensors do not present high variability, as their coefficient of variability is mostly below 0.1 (10%) for most of the events (Supplementary Fig. S1).

We identified two eruptive periods (A and B, Fig. 2a) happening at the time of recording, also in agreement with a previous study^20^. The eruption periods are characterized by sudden increase in tremor RMS seismic amplitude and number of volcanic explosions per day (Fig. 2a). Notably, average p-p pressure and pressure rate values were higher during these periods (Fig. 2a).

We focus only on the eruptive period A, ranging from July 14 to July 22 (Fig. 2a; red box). Eruption period B (Fig. 2a; gray box) presents signal artifacts (saturation) in the DDSS^21^ produced by large amplitude (>100 Pa) volcanic explosions and infrasound tremor, which could interfere with the detection, classification and analysis of the GR events (see Methods and Supplementary Fig. S2). The DDSS dynamic range set during this study is about $3 \cdot 10^{-5} s^{-1}$ due to acquisition parameters^21^ (see Methods: Data acquisition). This translates to a p-p strain rate amplitude limit of $6 \cdot 10^{-5} s^{-1}$.

To investigate the GR, we analyse the interaction between a single type of acoustic pressure input (explosions) and the different resulting deformation outputs in the ground along the fibre path (GR signals in DDSS). To isolate different types of acoustic inputs, we classify the explosions based on waveform similarity (WS; see Methods: Classification of Volcanic Explosions), a robust and simple approach, also applied to infrasound signals at Mt. Etna^22,23^. This classification method groups explosions into explosion families using the maximum cross-correlation (CC) value obtained between pair of events, and comparing it with a CC threshold (CC threshold of 0.88). The method also ensures that each explosion family contains at least two similar waveforms (see Methods: Classification of Volcanic Explosions)^24,25^.

In total, we identified 111 volcanic explosion families. Fig. 2b displays the first thirteen explosion families, with templates shown in Fig. 2c. Some explosion families such as 10 and 13 exhibit two distinct explosions in their templates (Fig. 2c), which is a product of classifying explosion windows containing two pulses with an occurrence is less than 2 seconds.

The classification algorithm sorts families in descending order by the number of explosions they contain; thus, explosion families 1 to 5 comprise 5683, 479, 221, 147, and 84 explosions, respectively, making up 78% of the 8394 explosions in the eruptive period A. Explosion families 6 to 111 comprises 15% of the total and only near 7% (528 explosions) remain unclassified.

Two craters were active during the eruptive period A. Back-azimuth estimates from the infrasound array (Methods: Detection of volcanic explosions) indicate that the NSEC crater (Fig. 1) produced most of the explosion signals. Due to the azimuthal coverage of the network, craters NEC and BN, which were active at this period with NSEC^26^, can not be differentiable. All explosion families are exclusively from NSEC, with exception of family 4, which could have its origin from BN, NEC and NSEC (Fig. 2b). Additionally, explosion signals from BN/NEC have generally less amplitude and longer periods than the ones coming from NSEC (Fig. 2b and 2c).

### Ground response observations

**Fig. 3:**
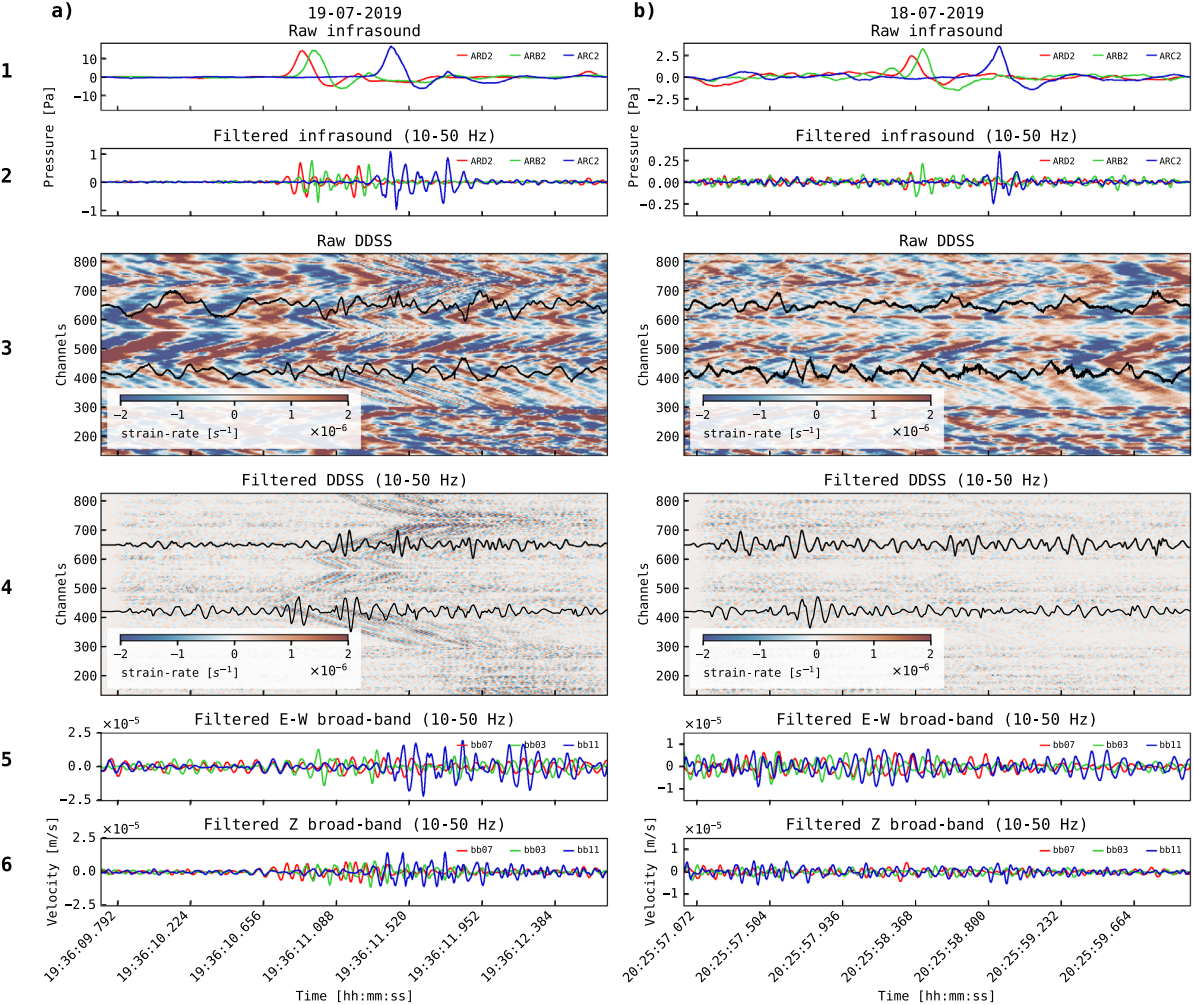
Two examples (**a** and **b**) of recorded volcanic explosions. The first row shows the raw infrasound signal recorded at three different infrasound sensors (ARB2, ARC2, ARD2). The second row shows the filtered signal between 10 and 50 Hz. The third row shows the corresponding GR event observed in raw DDSS data with two examples of DDSS signals at channels 420 and 650. The fourth row shows the same DDSS data of row third, filtered between 10 and 50 Hz. The fifth row is filtered (10 - 50 Hz) signals recorded on E-W component at three different broad-band seismometers. The sixth row shows the vertical (Z) component. The two explosions correspond to the same explosion family (explosion family 1), day (18-07-2019) and source (NSEC; back-azimuth estimations; see Methods: Detection of volcanic explosion). The explosion in **a** triggers a clear GR event, while in the explosion **b** the GR event is not clearly observable.

We analyse the ground response (GR) signals recorded in the DDSS due to the energy coupling of the volcanic explosions infrasound waves into the near-surface. The observed GR events are high-frequency signals (10–50 Hz) of short duration ($<1$ sec) recorded in the near-surface and linked to volcanic explosions. To build a GR event catalogue, a three-second window of DDSS data is selected, centred on the mean arrival time of the highest peak in the waveform associated with the explosion recorded across all infrasound stations (see Methods: Ground Response classification). Some GR event windows show clear GR signals across channels with 10–50 Hz frequency content, while others do not. For example, Fig. 3 compares the ground response differences between two volcanic explosions captured by the infrasound array. Both explosions originate from the New Southeast Crater (NSEC) and belong to the same explosion family (explosion family 1).

The first explosion example (Fig. 3a) shows an average peak-to-peak (p-p) pressure amplitude of 17 Pa, calculated from individual measurements across the infrasound array and filtered between 0.7 and 4 Hz. This explosion triggered a ground response above 10 Hz, observable in the unfiltered DDSS data (Fig. 3a, fourth row). The BB data also captured the ground response, observable with an applied 10–50 Hz band-pass filter (Fig. 3a, fifth row).

In contrast, the second explosion example (Fig. 3b) produced a p-p pressure amplitude of 3.4 Pa (filtered between 0.7 and 4 Hz), lower than the first explosion, but it did not generate a clearly observable ground response (Fig. 3b). However, in this case, the DDSS record indicates a possible ground response in a specific range of channels (700–800) compared to the other channels (Fig. 3b, fourth row). More examples of volcanic explosions can be seen in Supplementary Fig. S3 to S7, and associated GR events in Supplementary Fig. S7 to S10.

High-frequency content is also present in the explosion signals (Fig. 3). Filtered signals between 10 and 50 Hz (Fig. 3, second row) reveal emergent high-frequency waves occurring simultaneously with the primary low-frequency signal of the volcanic explosions (Fig. 3, first row).

### Ground response classification

Each explosion might produce a resolvable GR signal at multiple channels. The produced GR signal could differ from channel to channel due to different variables, such as: cable segment orientation respect to craters, changes in the local near-surface properties (temporal and spatially), or amplitude of the explosion signal. Therefore, to find any unbiased relationship between explosions (taken as input; see Results: Volcanic explosions catalogue & classification) and the induced GR signals (taken as output), we classify the GR signals independently from the explosions.

To account for spatial and temporal variability of the GR signals, we modified the waveform similarity (WS) algorithm from the explosion classification (see Methods: Ground response classification) in order to relate GR waveforms, both temporally and spatially. The GR classification required two rounds: a temporal classification (along the time axis for each channel) and a spatial classification (across templates for each channel). We validate the GR classification method using constructed synthetic GR data to recreate the complexity of temporal and spatial variation in GR signals (see Methods: Ground Response (GR) classification).

Processing continuous DDSS data spanning entire days or weeks demands substantial computational resources due to the high temporal and spatial resolution of the recordings. Additionally, strain rate values on individual channels can vary significantly due to the strong influence of the local media^27^. However, as part of the preprocessing routines, the used DDSS interrogator performs a spatial averaging over the channels covering a segment equal to the gauge length value (10 meters)^28^. Therefore, we selected channels of the GR event windows every 10 meters (from channels 134 to 825) to limit the analysis to independent measurements per gauge length.

The GR event classification identified 717 GR families. Unlike the explosion classification, the final order of families in the GR classification is not necessarily ranked by the number of members due to the two-round clustering process. Approximately 92% of the total waveforms across all GR events and channels remained unclassified.

**Fig. 4:**
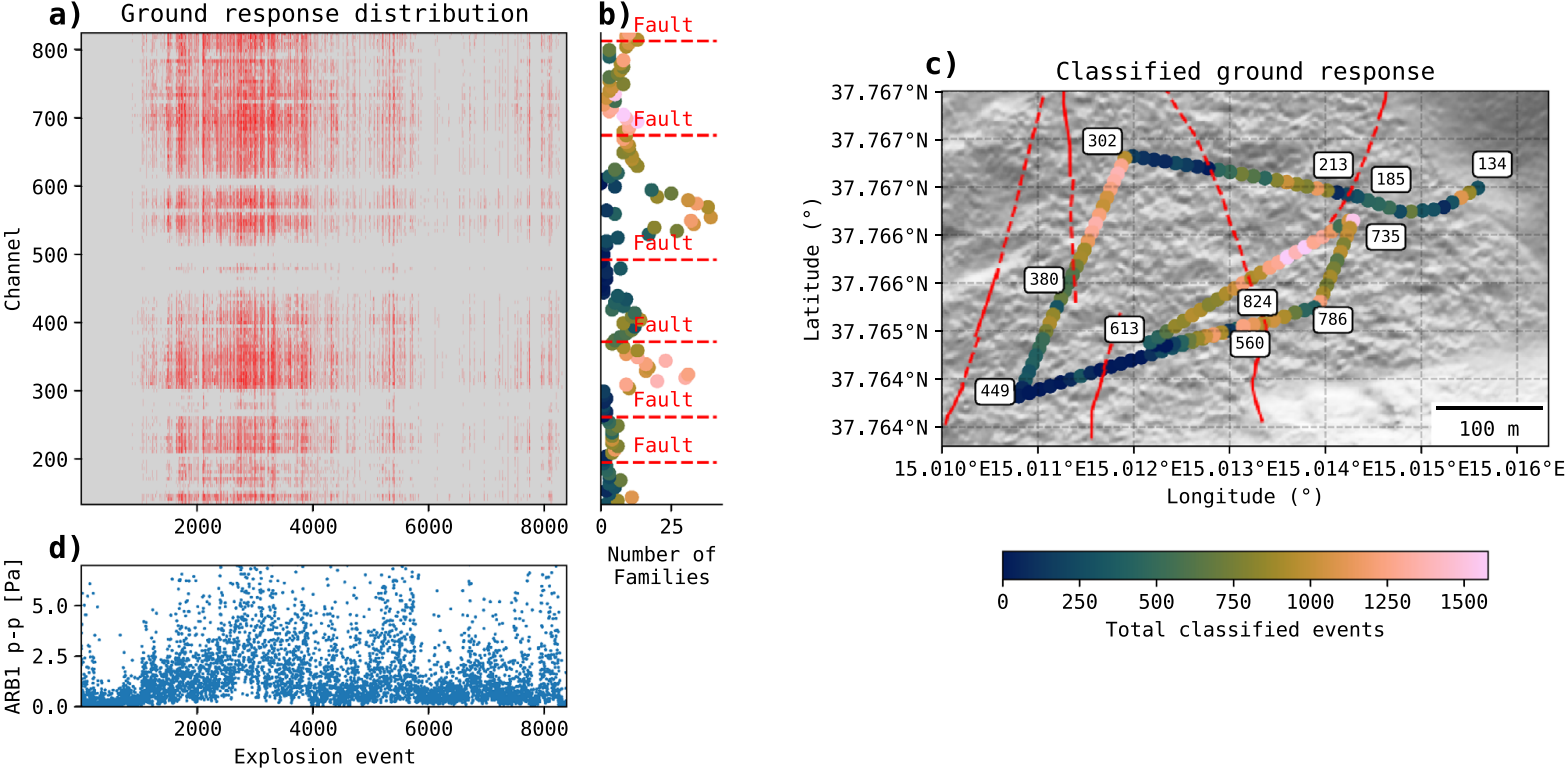
Obtained GR families from GR classification. **a)** Distribution of the obtained GR families across channels and for each explosion. Red colour represent classified GR event signals. Grey areas indicate GR event signals that were not classified (residuals). **b)** Number of GR families present at each channel. Fault crossed by the fibre optic cable are indicated in dashed red lines. **c)** Number of classified events per channel. Basemap done with Python and geospatial packages^45,47–49^. d) peak-to-peak (p-p) amplitude of events captures at station ARB1.

Fig. 4a shows the spatial (channels) and temporal (consecutive explosions) distribution of classified ground responses, with events becoming more frequent between explosion 1000 and 4500. Spatially, the number of GR families per channel (Fig. 4b) remains relatively constant across neighbouring channels, except where the fibre optic cable path changes, or at fault crossings (Fig. 4b) and connectors (e.g., Channel 560). Certain channels, such as those in ranges 302–380 and 613–735, display a higher number of classified GR events (Fig. 4c). Most of the GR families arise from explosions with high p-p pressure amplitude (Fig. 4d).

**Fig. 5:**
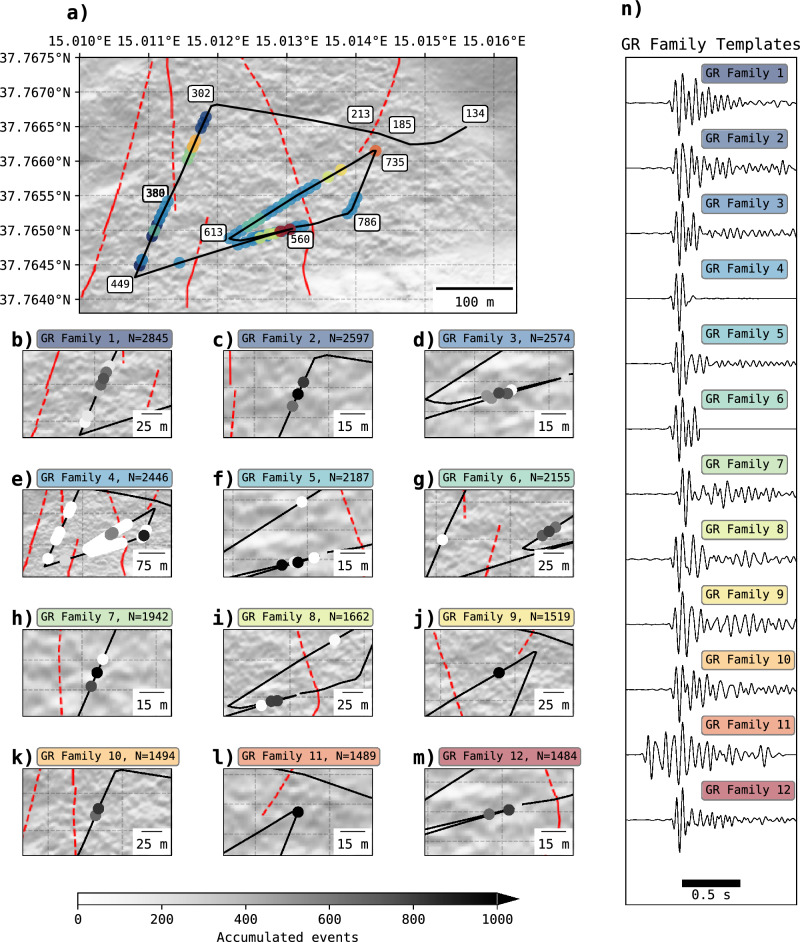
Spatial distribution of the first 12 GR families with the highest number of members. **a)** Overview map illustrating the presence of the first 12 GR families along the fibre optic cable (black curve) with distinctive colours for each GR family. Colours are connected to the labels of each GR family in subfigures **b - m)** and **n)**. **b - m)** Zoomed view showing the local distribution of each GR family and their occurrence (number of accumulated events) at each channel where they are present, indicated by colour scale (lower part). **n)** Obtained waveform templates (normalized amplitude) from GR classification for each of the 12 GR families. Basemap done with Python and geospatial packages^45,47–49^.

The GR classification reveals that GR families are localized within specific sections of the fibre optic cable. Fig. 5a-m illustrate that GR families with the most members are concentrated in nearby channels within particular regions of the fibre path not spanning more than 50 m, with exception of GR family 4. Additionally, most of the GR waveform templates have a similar signature at the beginning of the wave, and discrepancies later in the coda train (Fig. 5n).

### Strain rate and Pressure rate relation

The largest explosion family could induce more than one GR family from the ait-to-ground coupling. Therefore, we relate the peak-to-peak (p-p) pressure rate amplitudes from explosion family 1 measured in infrasound sensors with the p-p strain rate from the largest resulted GR signals measured in DDSS. The relation is observed for each GR family created by explosion family 1. Explosion family 1, being the biggest explosion family, represents already the 68% of the total amount of explosion events. Additionally, they are all originated from NSEC, and its members have the highest pressure amplitudes from eruption period A (Fig. 2b).

The GR classification distinguishes clear from unclear GR waveforms, ensuring only classified GR waveforms contribute to p-p strain rate values. This enables us to map GR event types within the possible elastic-mechanical states of the near-surface media.

**Fig. 6:**
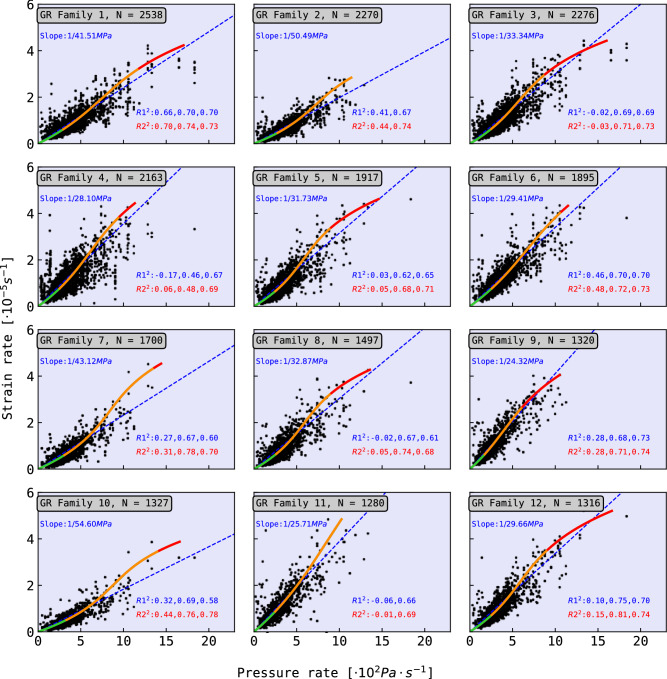
Strain rate of GR events with all channels associated to the GR families with the highest number of members vs. pressure rate of volcanic explosions from explosion family 1. Each plot presents a linear fitting curve (dashed blue line) and a 3rd order nonlinear curve (green-orange-red curve). The nonlinear curve represents the hyperleastic interpretation. The hyperplastic curve is composed by three stages: initial elastic behaviour (Stage 1: green curve), softening process (Stage 2: orange curve), and posterior stiffening (Stage 3; red curve). Each fitting curve has its $R^{2}$ score reported: R1 for linear fitting, and R2 for nonlinear. For each elastic stage, there is an individual $R^{2}$ score reported in order of the elastic stages.

In order to analyse the ground response per channel, Fig. 6 shows the relation between the p-p pressure rate from volcanic explosion of explosion family 1 and the strain rate amplitudes from the 12 biggest GR families previously shown in Fig. 5, at all channels. Each plot shows a linear fit (blue dashed curve) with a nonlinear fit as a third polynomial order. The nonlinear polynomial curve is divided into two or three segments, referred as elastic stages: Stage 1 (green), Stage 2 (orange) and Stage 3 (red). Each stage reports its $R^{2}$ value for each of the elastic stages of the nonlinear curve (R2) in comparison to the linear fitting (R1). The $R^{2}$ fitting score for a model between values $n \le i \le N$ is calculated as:

$$\begin{aligned} R^{2} = 1 - \frac{\sum _{i=n}^{N} (y_{i} - \hat{y_{i}})^{2} }{\sum _{i=1}^{N} (y_{i} - \bar{y})^{2} } \end{aligned}$$

where $y_{i}$ is the real value *i*, $\hat{y_{i}}$ is the predicted value from the model, and $\bar{y}$ is the mean value.

In most of the cases, the R2 score is higher than R1, showing that nonlinear fitting describe better the data than the linear fitting. The denominator value in the slope (Young Modulus) of the linear fit is reported at each subplot, ranging from around 27 to 55 MPa. These range of values are in agreement with literature regarding unconsolidated granular material^29^.

## Discussion

The spatial extent of GR families in PDC is limited, with GR waveforms spanning no more than 50 meters (Fig. 5). While family 4 appears in two distinct areas, far-adjacent channels record only 4–7 events, compared to 251–906 at the main sites. No gradual transition is observed between these areas. Main locations cover no more than five consecutive channels, consistent with the 10 meters gauge length and spatial decimation of DDSS data. This limited extent is attributed to the high cross-correlation (CC) threshold (0.87), which accounts for both the main GR signal and its coda. At lower CC thresholds (0.75, Supplementary Fig. S11), classification is dominated by the main pulse, leading to merged families and broader spatial coverage. This suggests that the 10 meters gauge length averages GR waveforms across neighbouring channels, while GR varies over <10 m. Capturing high-frequency GR more effectively would require a shorter gauge length (e.g., 2 meters) and higher sampling rates to preserve strain rate resolution and enhance dynamic range. Greater dynamic range would have prevented DDSS signal saturation, allowing detection of higher strain rates from strong explosions , but compromising the sensitivity to small GR events^21^.

Despite that the different segments of the cable align towards different craters, explosions from one distinctive crater can generate GR events across most of the fibre segments. Fig. 6 demonstrates that explosion family 1 is able to induce many GR events from the 12 biggest GR families. Additionally, Fig. 5 show that the 12 biggest GR families occupy specific fibre locations, but together the cover most of the cable segments, except for perpendicular segments to the craters (channels 185-302) or segments where ground composition is not scoria but other lithic fragments (channels below 155).

We observe temporal variability in ground response (GR) at specific locations. As shown in Fig. 6, a single explosion family can produce distinct GR signals such as GR families 7 and 10, or 5 and 12 (Fig. 5), depending on the site. This variation, despite identical explosion input, likely results from (1) changes in near-surface ground properties and (2) differences in the explosions’ high-frequency content. Of these, variations in ground properties appear to be the main factor. Fig. 5n shows that GR family differences lie primarily in the coda, while the initial pulse remains similar, suggesting consistent high-frequency coupling. However, due to the absence of environmental data (e.g., humidity, temperature, rainfall), we cannot confirm the influence of ground property changes. The local scoria is highly angular^15^, which enhances interlocking and reduces humidity sensitivity^30^, but its high porosity (76–83%)^31^ allows it to retain rainwater, causing observed ground wetness. No further patterns emerge when comparing diurnal/nocturnal hours or days after the second eruption onset (Supplementary Fig. S12, S13).

Regarding the mechanism responsible for the GR events, we rule out shearing between grains as the source. In granular materials, shear deformation can generate acoustic emissions through jamming, collisions, and frictional slips between particles, releasing strain energy as high-frequency elastic waves^32^. These emissions typically occur above 10 kHz^32^, which exceeds our sampling limit (1 kHz) and the observed frequencies for GR events (10-50 Hz).

Previous research attributed the high-frequency GR events at PDC to resonance effects related to the scoria layer’s thickness overlying the volcanic bedrock^15^. To evaluate this, we computed the horizontal-to-vertical spectral ratio (H/V) using the broadband (BB) stations to identify frequency peaks, interpreted as indicative of local site amplification^10,33^. We used 24 min windows of ambient noise (overlapped) over an entire day (06.08.2019) where seismic amplitude was at its lowest (Fig. 2a). The horizontal component for the H/V ratio was calculated as the quadratic mean of the north and east components^34^.

**Fig. 7:**
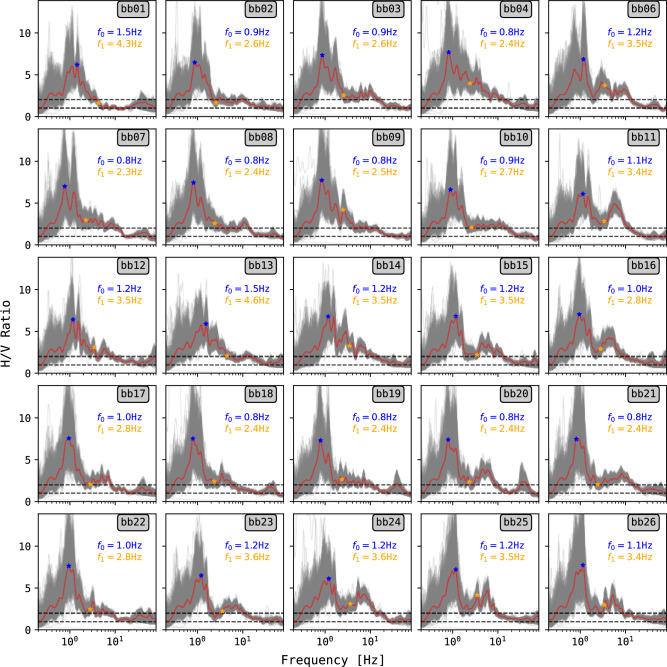
H/V curves on BB stations obtained on the 06.08.2019, the day with the lower RMS seismic amplitude (Fig. 2). Red curve shows the average from individual H/V curves (gray). Black dashed lines indicate H/V ratios of 1 and 2 for reference. Prominent frequency peaks can be observed between 0.8 and 1.2 Hz. Fundamental mode and theoretical first order harmonic are indicated by a blue and orange star, respectively. High-frequency peaks (<10 Hz) rarely overcome a ratio of 2.

The H/V curves show prominent resonance frequency peaks between 0.8 and 1.5 Hz with ratio values above 5 , associated with the fundamental mode of the scoria layer^35^. We interpret the resonance peaks as reliable since the follow the reliability criteria^36^: 1) there should be a frequency *f* related to $$f_{0}$$ as $$f_{0}/4< f < f_{0})$$ and $$f_{0}< f < 4 f_{0}$$ such that its H/V amplitude *A*(*f*) satisfies $$A(f) / A(f_{0}) > 2$$, and 2) $$A(f_{0}) > 2$$. Some of the resonance peaks are not narrow but rather broad in bandwidth, likely due to high local variability of the scoria layer thickness^35^. Higher-frequency peaks associated to harmonics of the fundamental mode might be observed, following the relation $$f_{n} = f_{0}(2n+1)$$, where *n* is the harmonic order, and $$f_{0}$$ is the fundamental mode^10,12^. BB stations like bb06, bb09, bb25 might exhibit first-order harmonics below 10 Hz (Fig. 7, orange dots). Nevertheless, we take cautions in this interpretation as the harmonic peaks barely fulfil the reliability criteria. At even higher frequencies corresponding to GR events (10 - 50 Hz), the H/V ratio decreases (Fi.g 7) and peaks in such frequency range do not fulfil the reliable criteria. Therefore, GR events are not due to a resonance of the scoria layer.

Given the fundamental mode (0.8 - 1.5 Hz) associated to H/V analysis, and using a shear wave velocity of 165 m/s^15,35^, the thickness of the excited layer would range from 27 to 51 meters, in agreement with values using other methods^15,35,37^. Assuming GR frequency values (10 - 50 Hz) it would correspond to 4 to 0.8 meters of layer thickness. However, models in literature^15,35,37^ do not resolve strong impedance contrasts at those depths, necessary for a clear resonance peak^10,36^.

The high-frequency content from explosions is amplified by the near-surface scoria material, unrelated to layer resonance. While in raw infrasound signals (time and frequency domains) the high-frequency components cannot be clearly recognized, in DDSS data they are resolvable (Fig. 3). GR event frequencies remain similar across channels during a single explosion. At a layer resonance situation, frequencies would show spatial variability across channels, as PDC exhibits spatial variability in the scoria layer thickness^15,17,35^. No transfer from the low frequency components to the high frequency is observed. Thus, GR events are likely influenced by other scoria properties, such as particle size distribution, rather than layer thickness. Additionally, reflections of GR waves due to nearby structures have been previously reported^15^. The presence of reflections indicate that GR waves are not stationary, supporting the notion that GR events are not associated with scoria layer resonance. No specific pressure threshold is expected for triggering a GR event, as it depends both on the input amplitude, and local scoria properties that may make GR signals more detectable at certain locations (Fig. 3b).

In terms of mechanical behaviour, two models are contemplated for the scoria material. The first model describes linear elasticity (Fig. 6, blue dashed line), where the relationship between strain rate and pressure rate follows a linear trend. In this case, the inverse of the slope corresponds to the Young’s modulus. The second model is a nonlinear elastic response described by a third-order polynomial, segmented into either two (Fig. 6, green-orange curves) or three (Fig. 6, green-orange-red curves) elastic stages. Due to the nonlinearity, the elastic modulus varies along the curve, and no single slope value can be defined.

The boundaries between the stages of the nonlinear model are defined at the points where the curve’s slope matches that of the linear model, which serves as a reference. Stage 1 (green) exhibits a near-linear relationship between pressure rate and strain rate, consistent with linear elasticity. Stage 2 (orange) is characterised by a steep increase in strain rate with minor changes in pressure rate, reflecting material softening and the onset of nonlinear behaviour. At higher pressures, some curves transition into Stage 3 (red), where the strain rate stabilises despite increasing pressure, indicating material stiffening. This progression through three elastic stages is indicative of hyperelastic behaviour, typical of unconsolidated sediments^3^.

The hyperelastic model (second model) usually provides a better fit to the data than the linear model (first model) in terms of higher $R^{2}$ values for individual stages (Fig. 6). Although, the $R^{2}$ values are no significantly different between the two models, and so they cannot be distinguished based on current analysis. Furthermore, the number of explosion events with sufficiently high pressure rate values is limited, which restricts the evidence supporting the stiffening phase of the hyperelastic model. Explosions from Eruption Period B (Fig. 2a) display high pressure rates but result in saturation in the DDSS recordings. As a result, both models in this case are considered capable of describing the mechanical behaviour of the scoria at PDC. However, further modelling and the acquisition of additional explosion data using shorter gauge lengths would be necessary to draw firmer conclusions about the scoria’s mechanical behaviour.

Typically, wave distortion, such as frequency shifts, is expected in materials under static loading, or induced hysteresis processes^38,39^. Nevertheless, GR families are observed across all the three elastic stages of hyperelasticity, implying that the observed high-frequency ground response is nonlinear.

The elastic ground response of the near-surface granular media (scoria) at PDC (Mt. Etna) can be described as linear or hyperelastic. In case of hyperelasticity, softening and stiffening processes in the scoria arise due to acoustic coupling of volcanic explosion waves from volcanic explosions into the ground. On unstable slopes, such as those in the complex structures of Mt. Etna, landslides could potentially be triggered if volcanic explosions induce significant softening of the scoria material. This risk is heightened if the slope angle approaches the friction angle of the scoria (approximately $23^{\circ }$)^15^. Nevertheless, for the acquired dataset, no firm conclusion can be stated on which of the models fully describes the material.

The high-frequency ground response (GR events) observed with DDSS does not correspond to resonance effects of the scoria layer thickness. However, we interpret the GR events as a nonlinear site amplification of high-frequency components of volcanic explosions upon interaction with the near-surface scoria. The explanation behind this phenomenon might be related to the mechanical interaction between particles, which needs to be understood in depth using modelling and laboratory experiments. Even when explosions generate high-pressure waves, a ground response amplification is not observed unless the high-frequency components possess sufficiently large amplitudes (greater than 1 Pa peak-to-peak) and exhibit emergent characteristics with clear onsets.

## Methods

### Data acquisition

The multi-parametric network was installed in Piano delle Concazze (PDC), a large flat area of non-consolidated scoria deposits. The area stands at 2800 m.a.s.l. in the eastern flank of Mt. Etna^17^. The network recorded from 1st of July until 23th of September, 2019. The network comprises the following sensors:

- 26 Trillium Compact 120 s BB seismometers (from Nanometrics) distributed over the study area (PDC), with a inter-station distance of around 70 meters to create a dense seismic array (Fig. 1). Sensors were placed 40 cm beneath the surface, and connected to a digitizer (DATA-CUBE from DiGOS GmbH) in order to sample at a rate of 200 Hz.
- 9 Boise State University (BSU) infrasound sensors deployed at the surface at defined places over the fibre optic cable path (Fig. 1). The sensors recorded using the same digitizer and sampling rate (200 Hz) as the BB sensors.
- A 1.5 km fibre optic cable, buried 20 cm below the surface, laid out in several straight paths to form a triangular shape (Fig. 1). This geometry enhances the longitudinal sensing sensitivity of the fibre, as certain sections align with specific craters locations. The cable contains 12 individual fibres, one of which was connected to an iDAS interrogator unit (from Silixa Ltd.) at the nearby Pizzi Deneri Observatory for DDSS measurements. The iDAS collected data over the same period as the broadband (BB) and infrasound sensors, with a sampling rate of 1000 Hz, a gauge length of 10 meters, and a virtual sensor (channel) spacing of 2 meters, yielding 824 channels along the fibre (more acquisition parameters in Supplementary Table S1).

### DDSS channels georeference

Geographical positions of channels along the PDC-deployed cable section was obtained in a previous study^21^. Jumps were made at precise GPS-measured locations, primarily at the endpoints of each straight cable segment^21^. In the DDSS data, the channel showing the earliest arrival of each tap signal was assumed to be co-located with the tap location. Channels between located points were linearly distributed along each straight path. A validation was carried out by comparing the derived channel spacing with the acquisition setup, and accepted if the derived channel spacing is within a 20% error margin from the actual channel spacing.

### Detection of volcanic explosions

To detect volcanic explosions, we calculated the standard deviation (SD) over continuous infrasound data at each sensor, filtered between 0.7 and 4 Hz, to construct a characteristic function. The SD is defined as^23^:

1 $$\begin{aligned} SD^{2} = \frac{1}{n-1} \sum ^{n}_{i=1} (x_{i} - x_{m})^{2} \end{aligned}$$

where *n* is the number of samples in a defined time window, $x_{i}$ is the signal value of the $i^{th}$ sample and $x_{m}$ is the mean signal value within the window. SD is computed over 1-second windows with no overlap between windows. Two threshold values were set to define the start and end of an explosion when the SD function exceeds or drops below these thresholds.

To remove false detections or those inconsistent across the array, we applied a similarity threshold criterion using cross-correlation (CC) between detected explosions from each pair of sensors in the array. CC was computed over 2-second windows with a 1-second overlap, and explosions not meeting the threshold were excluded from the final catalogue.

Volcanic explosions show coherent arrivals across the infrasound array, with varying arrival times. We assumed plane wave arrivals across the array since the infrasound deployment area is no more than 0.3 by 0.3 km, and located 2.1 km away from the craters. Under this information and assumption, We performed a least square inversion^40–43^ in the form $d = Gm$, where *d* is the vector of arrival time delays between sensor pairs, *G* is the matrix of N-S and E-W distances between sensors, and *m* is the model vector. By minimizing the sum of squared errors, *m* is retrieved as $m = (G^{T}G)^{-1}G^{T}d$, which yields N-S and E-W slowness vectors. From these, we derived the back-azimuth and apparent velocities for each explosion, allowing us to identify the source crater.

### Classification of volcanic explosions

To understand the nonlinear ground response and identify the factors that may contribute to it, it is essential first to characterize the infrasound pulse from volcanic explosions as it couples into the ground. Given the extensive catalogue of explosions, we implemented an automatic classification approach based on waveform similarity (WS)^22–25^ in order to differentiate and characterize different types of explosions.

Using data from infrasound sensor ARB1, which has the highest signal-to-noise ratio (SNR), we computed a correlation matrix composed by the maximum cross-correlation (CC) scores achieved between each pair of explosion waveforms. This matrix served as the basis for explosion classification, which proceeded in two main steps: (1) selecting the row with the greatest number of elements (explosions) that meet or exceed a specified CC threshold, thereby grouping these elements into an explosion family, and (2) removing classified explosions from the matrix, reducing it row-wise and column-wise. These steps were repeated until the matrix consisted solely of unclassified explosions with scores below the threshold. Explosion families represented by only a single explosion were discarded and left unclassified. The outcome of this process are families of waveforms that share similarities in their shape. All individual waveforms of the events belonging to each respective family are stacked to form waveform templates.

Setting an appropriate CC threshold is a persistent challenge in automatic classification and machine learning studies, as it often requires manual selection rather than automation^22,23,44^. To determine the optimal threshold, we evaluated the classification algorithm across a range of CC thresholds and assessed their influence on classification outcomes (Supplementary Fig. S14). For each CC threshold, we calculated the number of unclassified explosions and achieved explosion families, looking for sudden changes that would indicate either an inflection point (the ”elbow” method) or a local maximum, helping to identify an ideal CC threshold. The final CC threshold was chosen based on these analyses, supplemented by visual inspection of the resulting explosion family templates and their individual waveforms, with alignment between waveforms and templates generally signalling successful classification.

### Ground Response (GR) classification

**Fig. 8:**
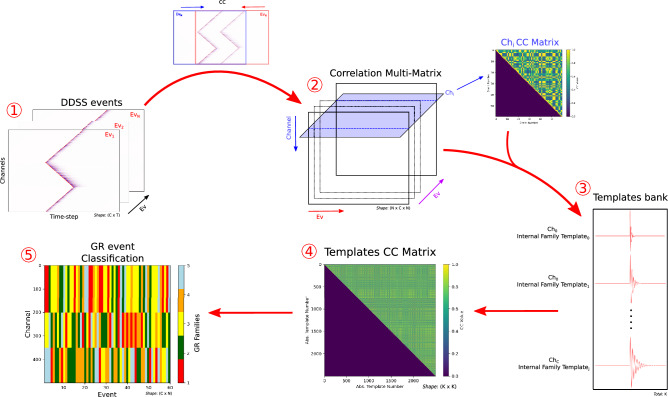
Schematic representation of the GR event classification algorithm. The algorithm works in two classification stages. The first classification stage (steps 1 to 3) is done by single 1D waveforms on each individual channel to create a template bank of all internal families from all channels. The second classification stage (steps 3 to 5) classify the obtained waveforms of the template bank into GR families, but keeping track of each waveform from each internal family obtained in the first stage.

To investigate the relation between the classified explosions in infrasound and the high-frequency ground response (GR) along the fibre, we classify the GR events recorded on DDSS and associated with the explosions. We define a GR event as the group of DDSS signals recorded across a range of channels of interest, and at a specific time-window. GR events are extracted using a 3 seconds-window spanning channels 134 to 825. The window is centred at the average arrival time of the infrasound signal detected by the infrasound sensors. Under this definition, a GR event is a matrix, where rows represent DDSS channels, and columns represent consecutive time-samples (Fig. 8, step 1). To enhance the detection of the nonlinear ground response, we bandpass-filtered the GR events between 10 to 50 Hz^15^ (Results: ground response observations).

We applied the same WS classification method used for the explosion classification. However, the method is adapted to scope the spatio-temporal distribution of the GR events, which means taking into account all waveforms across all channels, to enable both spatial and temporal classification. To validated the modified WS method, we created synthetic DDSS windows using sinusoidal waveforms with controlled frequencies and damping to simulate different onset and decay behaviours (Supplementary Fig. S15 and S16). The same evaluation criteria for optimal CC threshold is applied as with the explosion classification, separately for time (Supplementary Fig. 17) and spatial dimensions (Supplementary Fig. S18). Given the large amount of waveforms, computational efficiency is essential. Therefore, the following paragraphs describe the adapted WS algorithm and key concepts used in the GR classification.

Fig. 8 provides an overview of the classification workflow. We first cross-correlate each pair of GR events to form a correlation matrix, similarly to the explosion workflow. Here, the CC is performed by sliding one GR event over another along the time dimension, which corresponds to comparing signals registered at the same channel across different events. To accelerate this computation we use GPU-based matrix processing. The highest CC value obtained at each CC computation are stored in a three-dimensional correlation multi-matrix with dimensions $N \times C \times N$, where *N* and *C* are the numbers of GR events and channels, respectively (Fig. 8, step 2).

Each slice of the multi-matrix along the channel dimension *C* corresponds to a $N \times N$ correlation matrix for an specific channel $C_{i}$ (Fig. 8, blue square in step 2). For each channel $C_{i}$, a WS classification is applied. Similarly to explosion classification, families and their respective templates are obtained. Such families are referred as internal family for the specific channel $C_{i}$. Repeating this procedure for all channels yields a template bank of *K* internal family templates across the fibre (Fig.8, step 3).

To identify spatial patterns along the fibre, we then compare internal family templates across channels. While in theory a full CC matrix of size $(N \cdot C) \times (N \cdot C)$ could be computed, such task would require TBs of storage and memory. Instead, we perform a second round of WS classification using the templates bank. A correlation matrix of size $K \times K$ is computed between the templates, while keeping track of the original waveforms associated with each internal family (Fig.8, step 4). Internal family templates are then clustered into GR families. All individual waveforms associated with internal families are re-labelled according to their corresponding GR family (Fig. 8, step 5). This allows DDSS waveforms between channels to be clustered together within a GR family.

## Supplementary Information

Supplementary Information.

## Data Availability

The data used in this study is available by request to the authors (contact: pjousset@gfz.de) and will be put online at the GEOFON data repository after an embargo period of 3 years (2026).

## Code availability

The software and codes used are available upon request to the authors.
