# Supplementary material for: Insights in nonlinear ground response in volcanic environments from distributed dynamic strain sensing
Source: Sci Rep. 2025 Sep 24;15:32698. doi: 10.1038/s41598-025-20368-0 (PMC12460830; doi:10.1038/s41598-025-20368-0)
Supplement: Supplementary file 1 — Supplementary Information. [file 41598_2025_20368_MOESM1_ESM.pdf]

Understanding nonlinear ground response in volcanic environments using distributed dynamic strain sensing in Mt. Etna, Sicily.

Supplementary Material

May 26, 2025

1 Supplementary Figures

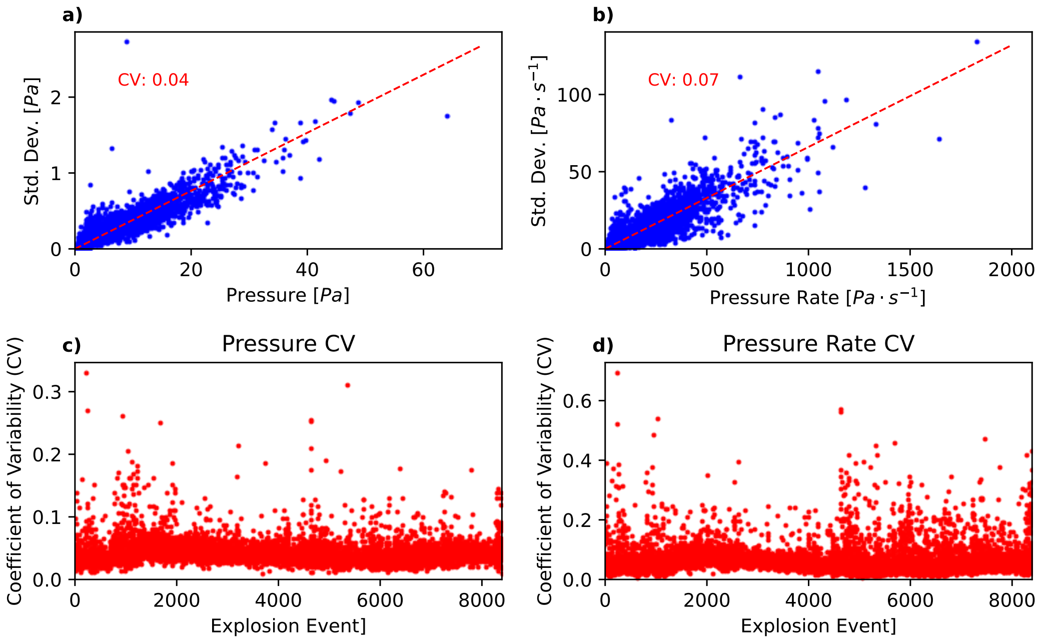

Figure S1: Error estimations for average p-p pressure and p-p pressure rate measurements at explosion events. Plots of p-p pressure ((**a**)) and pressure rate ((**b**)) amplitudes vs their respective standard deviations (std. dev.). Dashed red lines represent a linear regression where the slope gives the coefficient of variability (CV). Subfigures **(c)** and **(d)** show the CV for every value of average p-p pressure and pressure rate, respectively. Most of the values are below 0.1, indicating that the average p-p values are reliable.

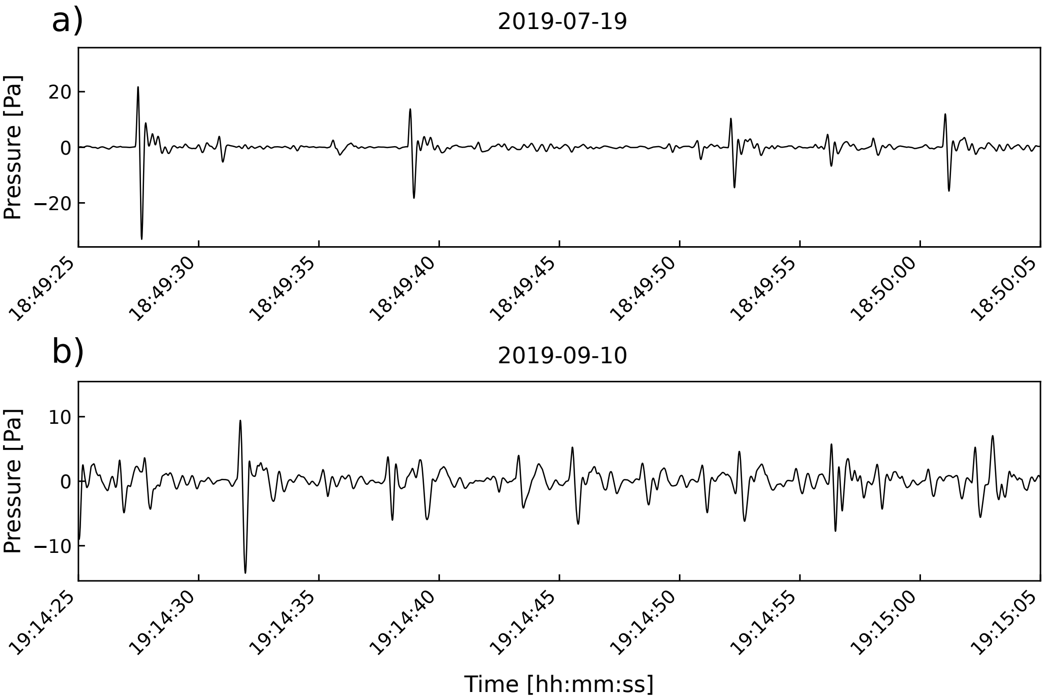

Figure S2: Example of explosion signals for infrasound sensor ARB1 on 2019-07-10 (eruption period A) and on 2019-09-10 (explosion period B). **a)** The explosion signals span more than 1.5 seconds between each other, which allows a single explosion window picking and validate the explosion detection criteria (see Methods). **b)** The explosions signals span less than 1.5 seconds between each other, which interferes with the explosion detection criteria.

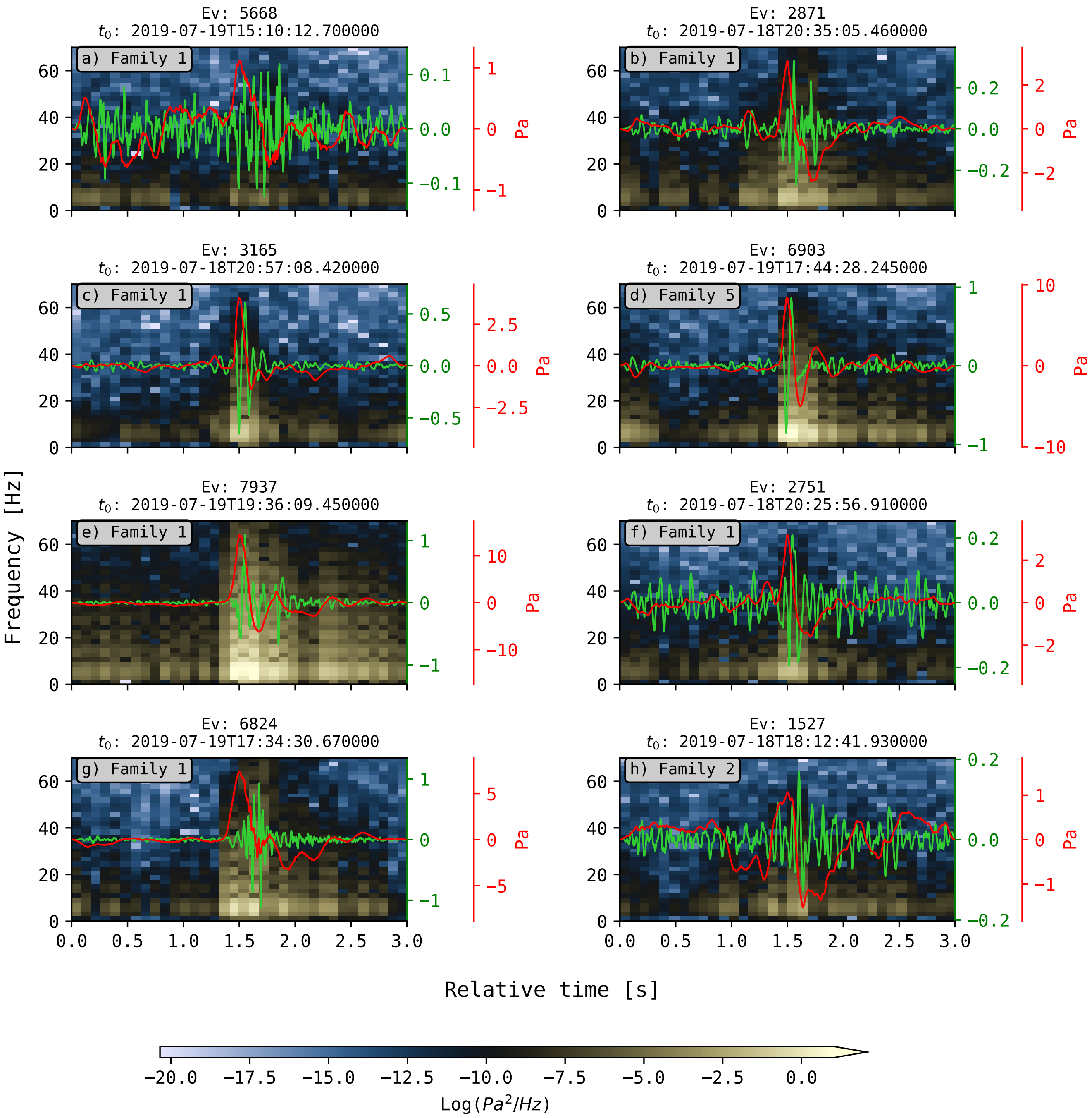

Figure S3: Random examples of volcanic explosion events recorded at infrasound sensor ARB2. The red curve is the raw waveform, and the green curve is the filtered waveform between 10 to 50 Hz. Both curves are on top of the respective spectrogram for the each event. Start time of the shown event windows is indicated on top, as well as the family which each one belong to after classification. Same events but in different infrasound sensors are also shown in Figures S4 and S5.

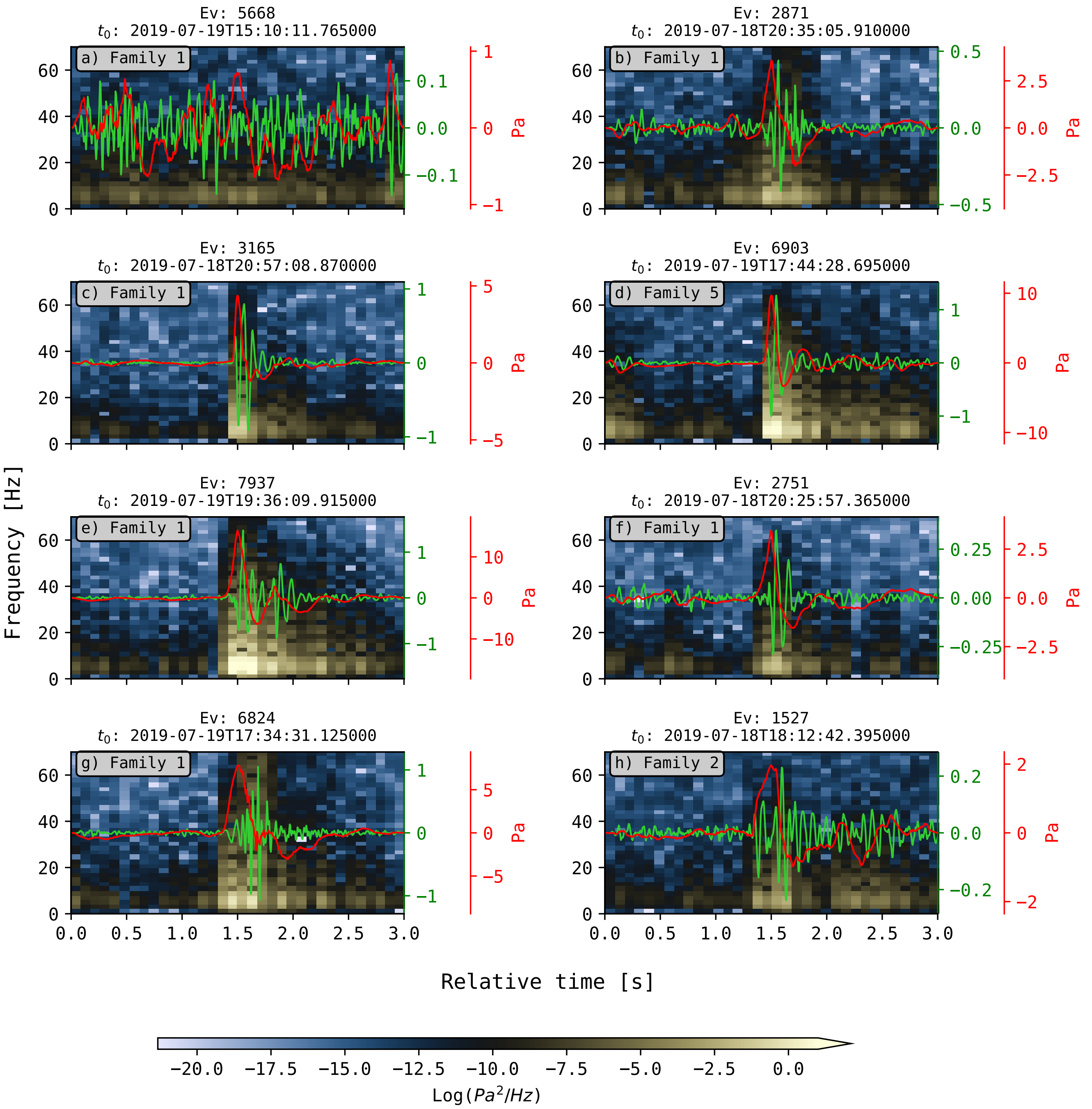

Figure S4: Random examples of volcanic explosion events recorded at infrasound sensor ARC2. The red curve is the raw waveform, and the green curve is the filtered waveform between 10 to 50 Hz. Both curves are on top of the respective spectrogram for the each event. Start time of the shown event windows is indicated on top, as well as the family which each one belong to after classification. Same events but in different infrasound sensors are also shown in Figures S3 and S5.

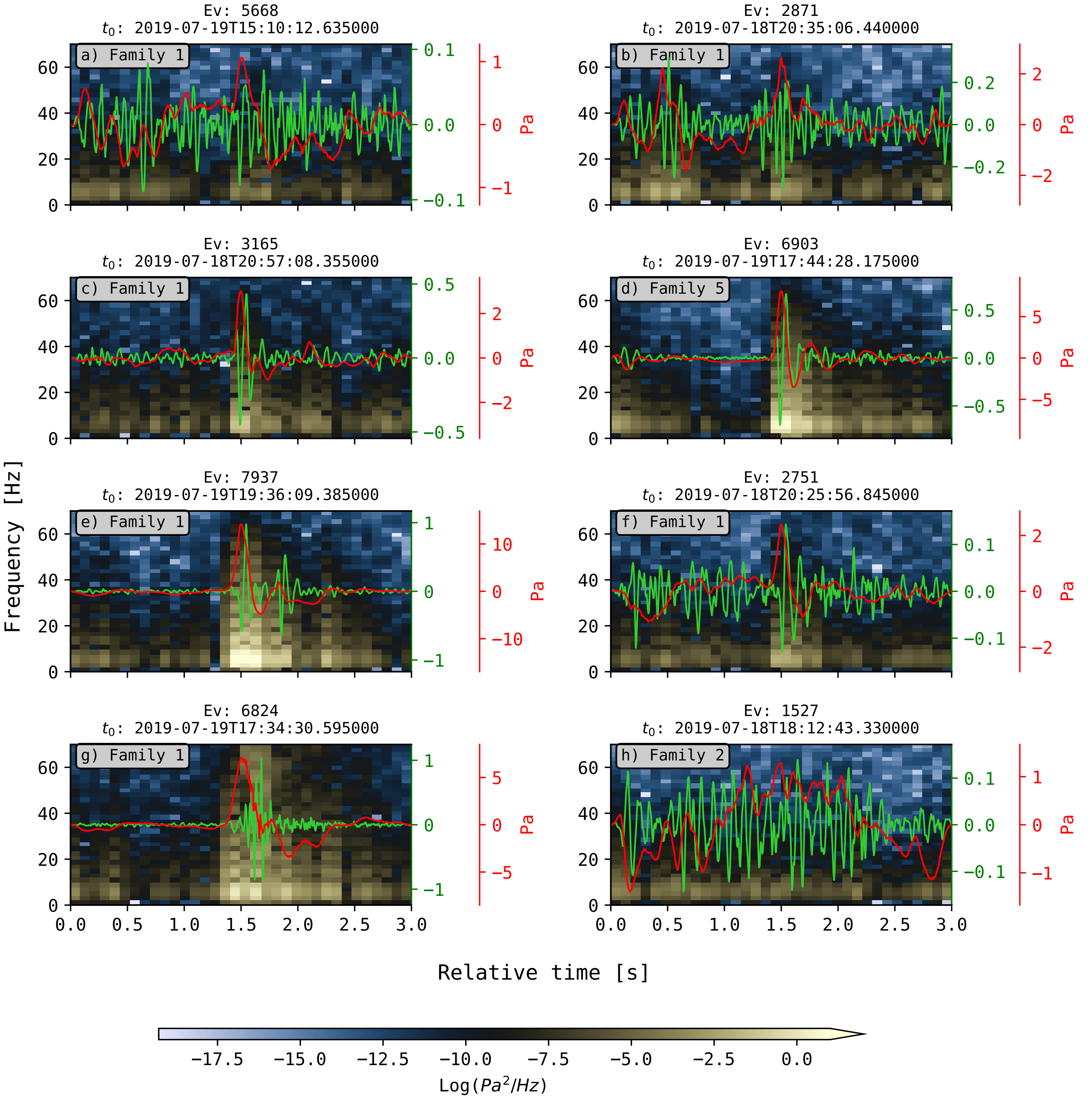

Figure S5: Random examples of volcanic explosion events recorded at infrasound sensor ARD2. The red curve is the raw waveform, and the green curve is the filtered waveform between 10 to 50 Hz. Both curves are on top of the respective spectrogram for the each event. Start time of the shown event windows is indicated on top, as well as the family which each one belong to after classification. Same events but in different infrasound sensors are also shown in Figures S3 and S4.

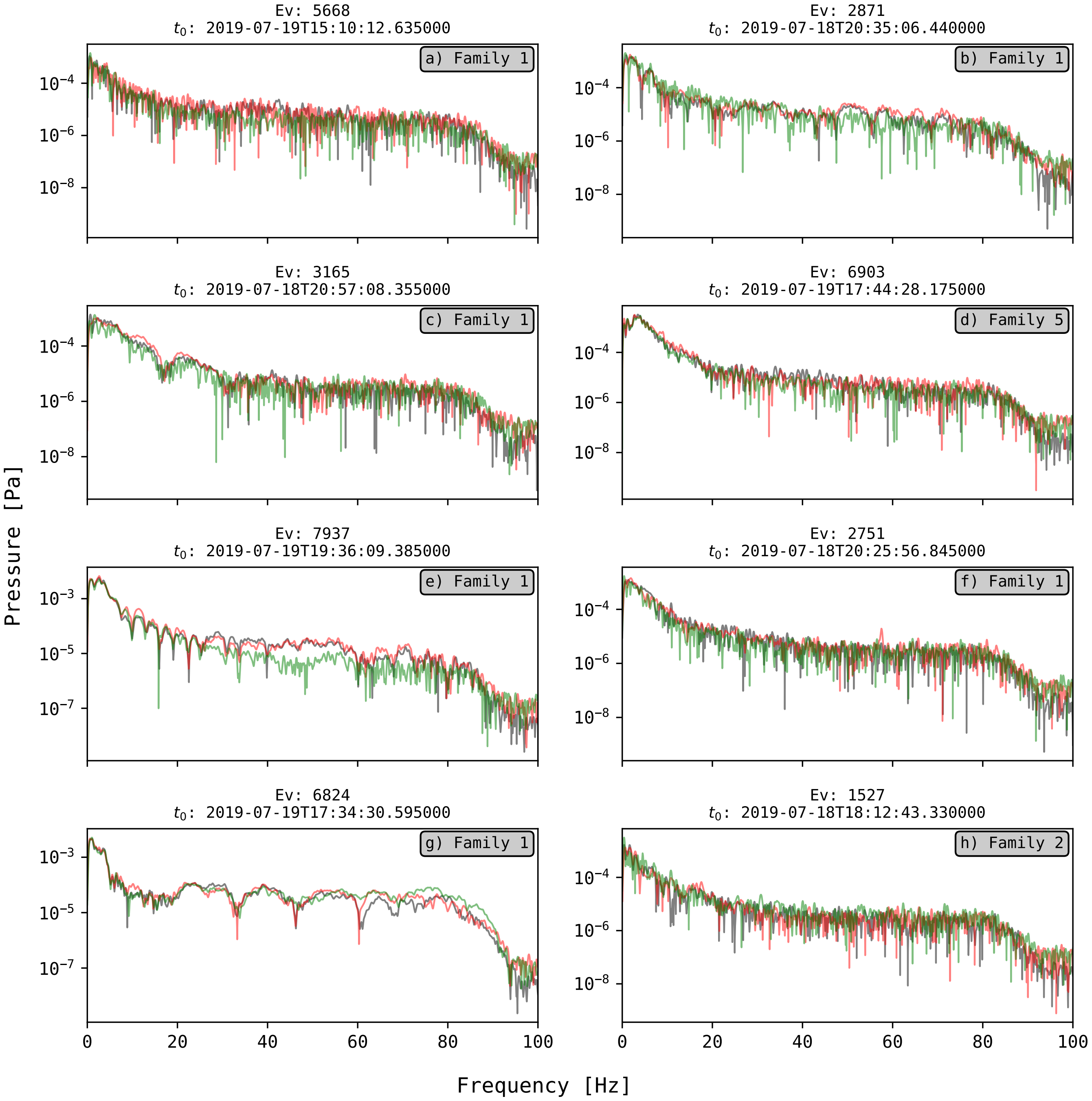

Figure S6: Spectral curves of random examples of volcanic explosion events recorded at infrasound sensors ARB2 (black), ARC2 (red), and ARD2 (green), as reference to Figure.

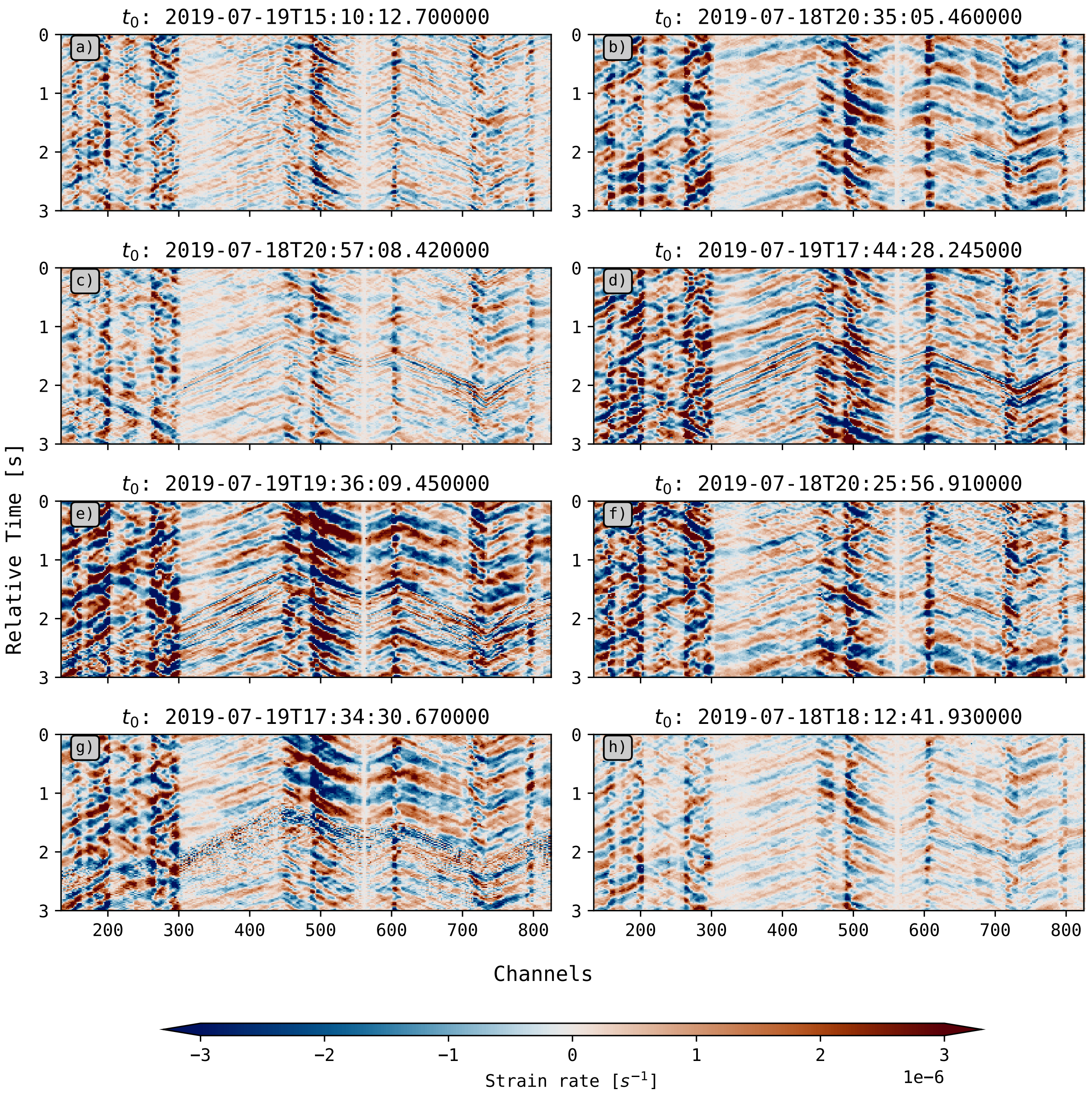

Figure S7: Raw DDSS ground response signals due to volcanic explosion events events shown in Figures S3, S4 and S5. Start time of the event window is indicated on top of each subfigure.

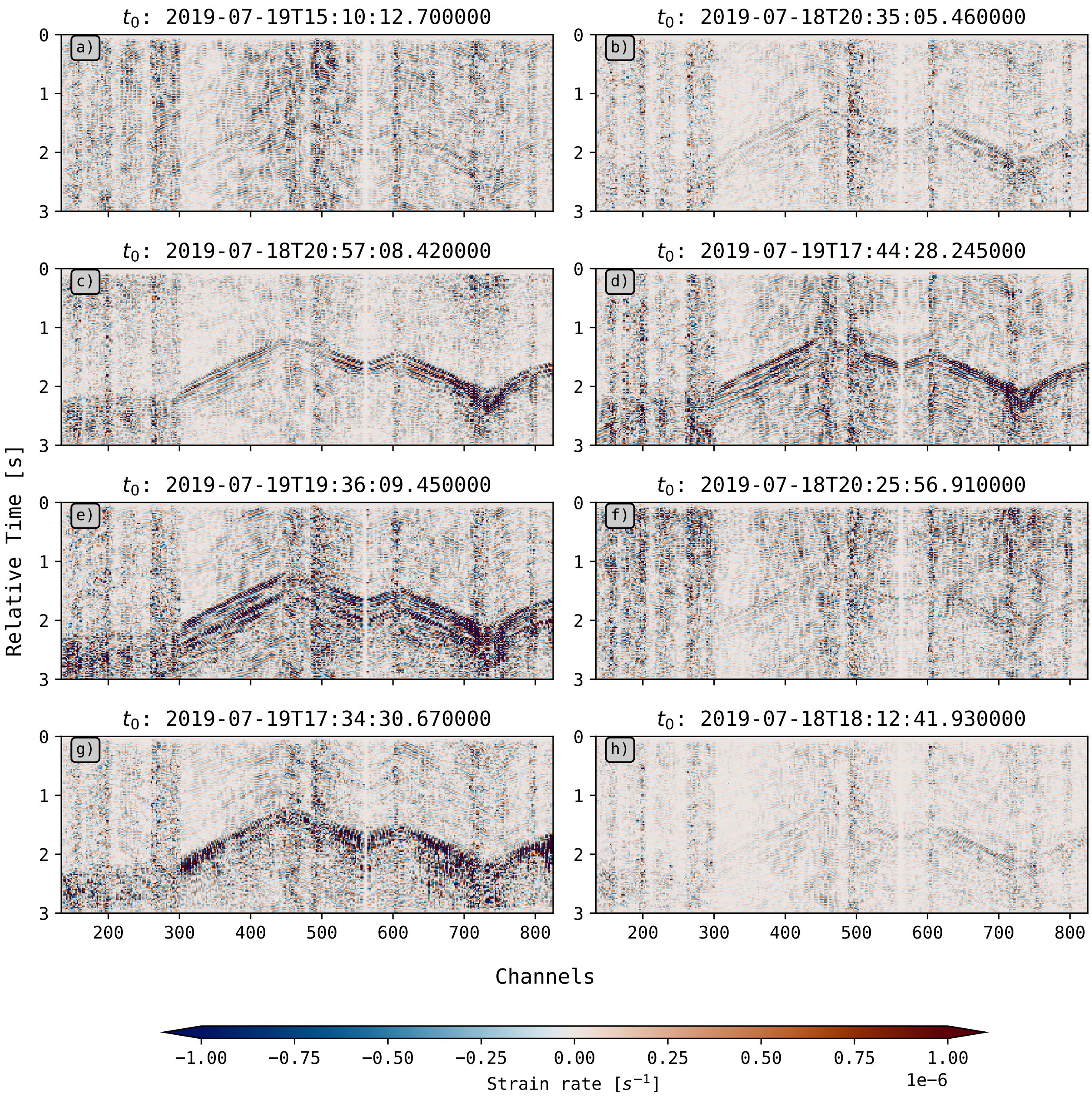

Figure S8: Filtered (10 - 50 Hz) DDSS ground response signals due to volcanic explosion events events shown in Figures S3, S4 and S5. Start time of the event window is indicated on top of each subfigure. High frequency arrival corresponding to the ground response are visible.

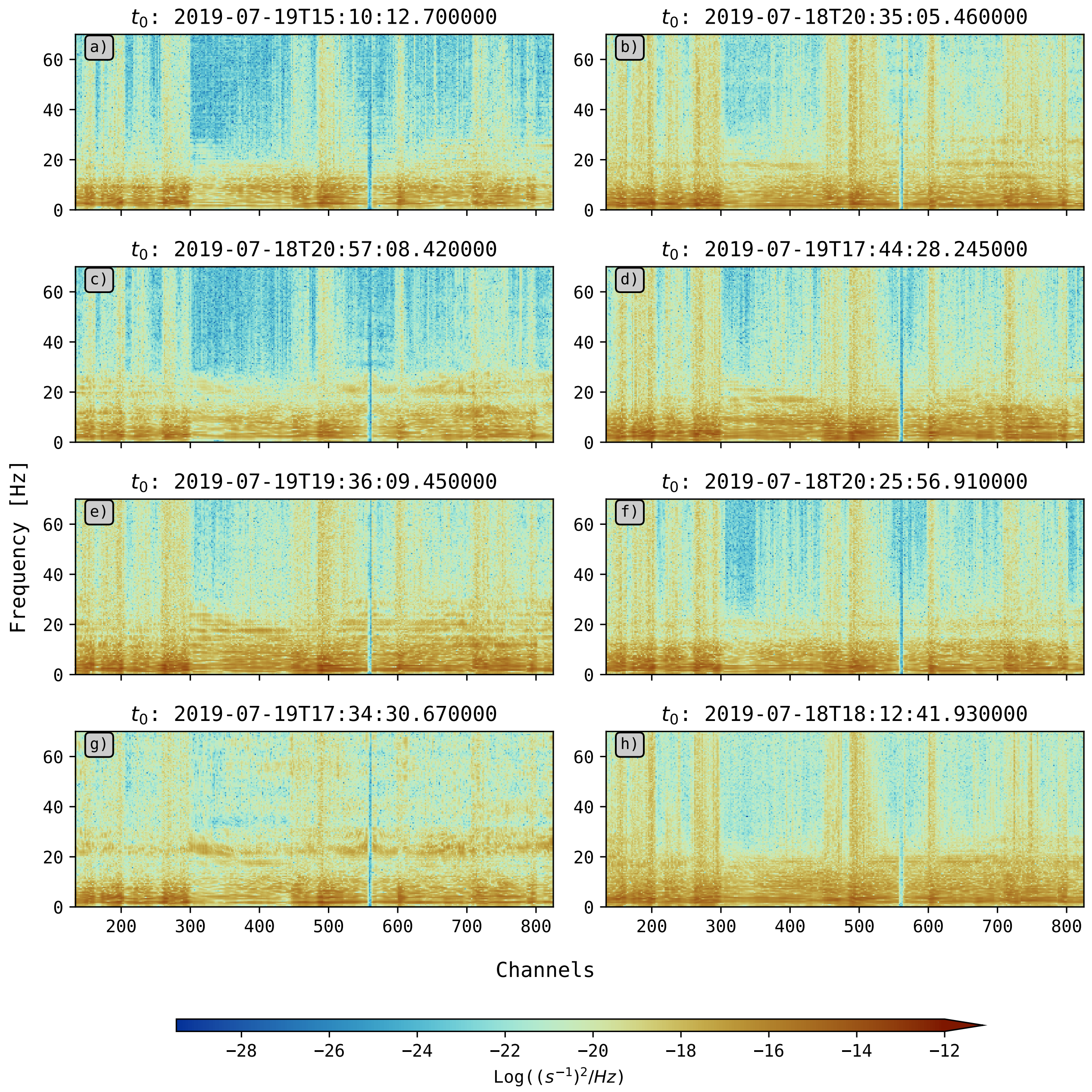

Figure S9: Spatial spectra of DDSS ground response signals due to volcanic explosion events events shown in Figures S3, S4 and S5. Start time of the event window is indicated on top of each subfigure. High frequency arrival corresponding to the ground response are visible.

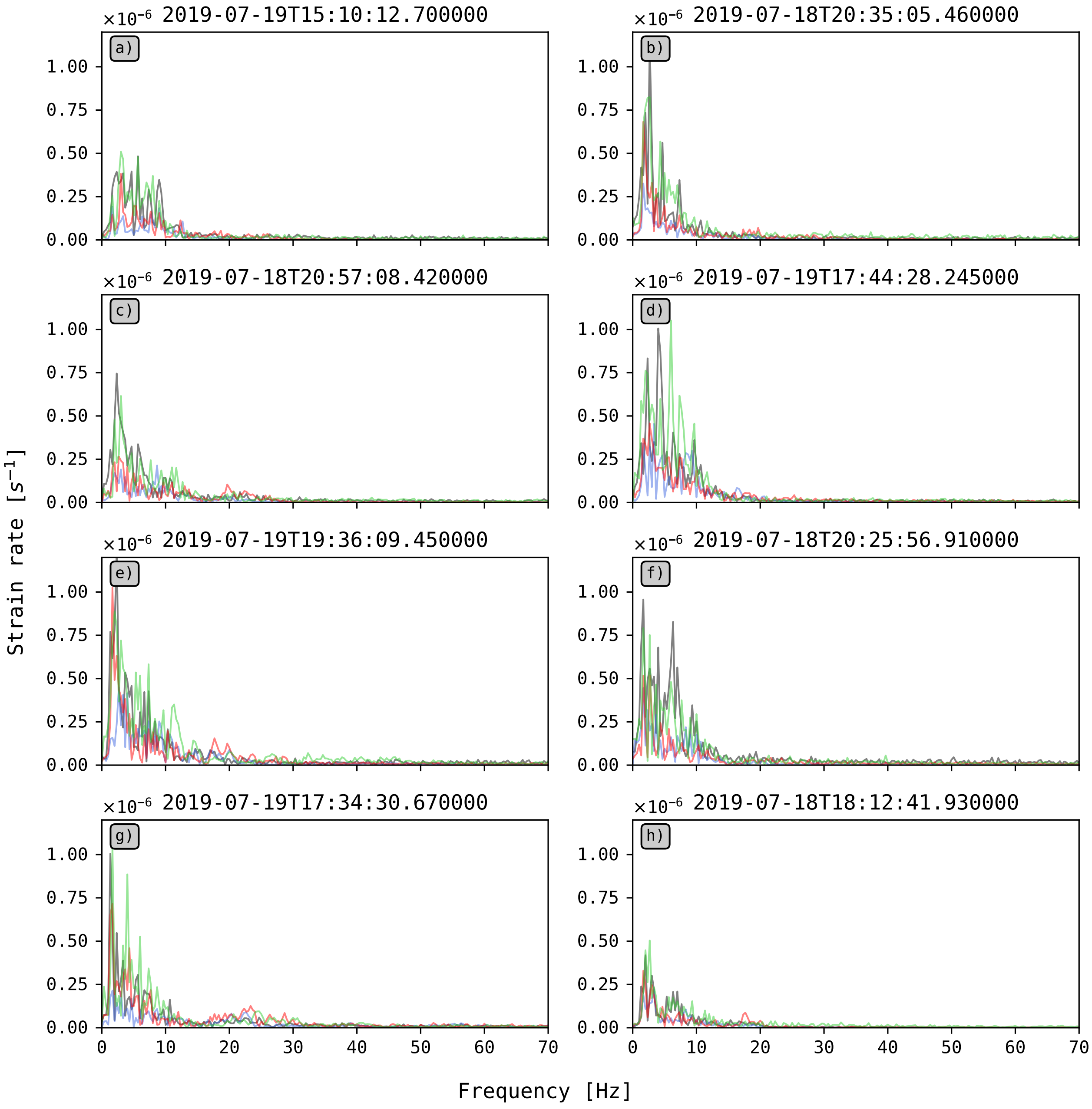

Figure S10: Channel spectral curves of DDSS ground response signals due to volcanic explosion events events shown in Figures S3, S4 and S5. Random channels where selected: 230 (black), 360 (blue), 670 (red), and 750 (green). Start time of the event window is indicated on top of each subfigure. High frequency arrival corresponding to the ground response are visible.

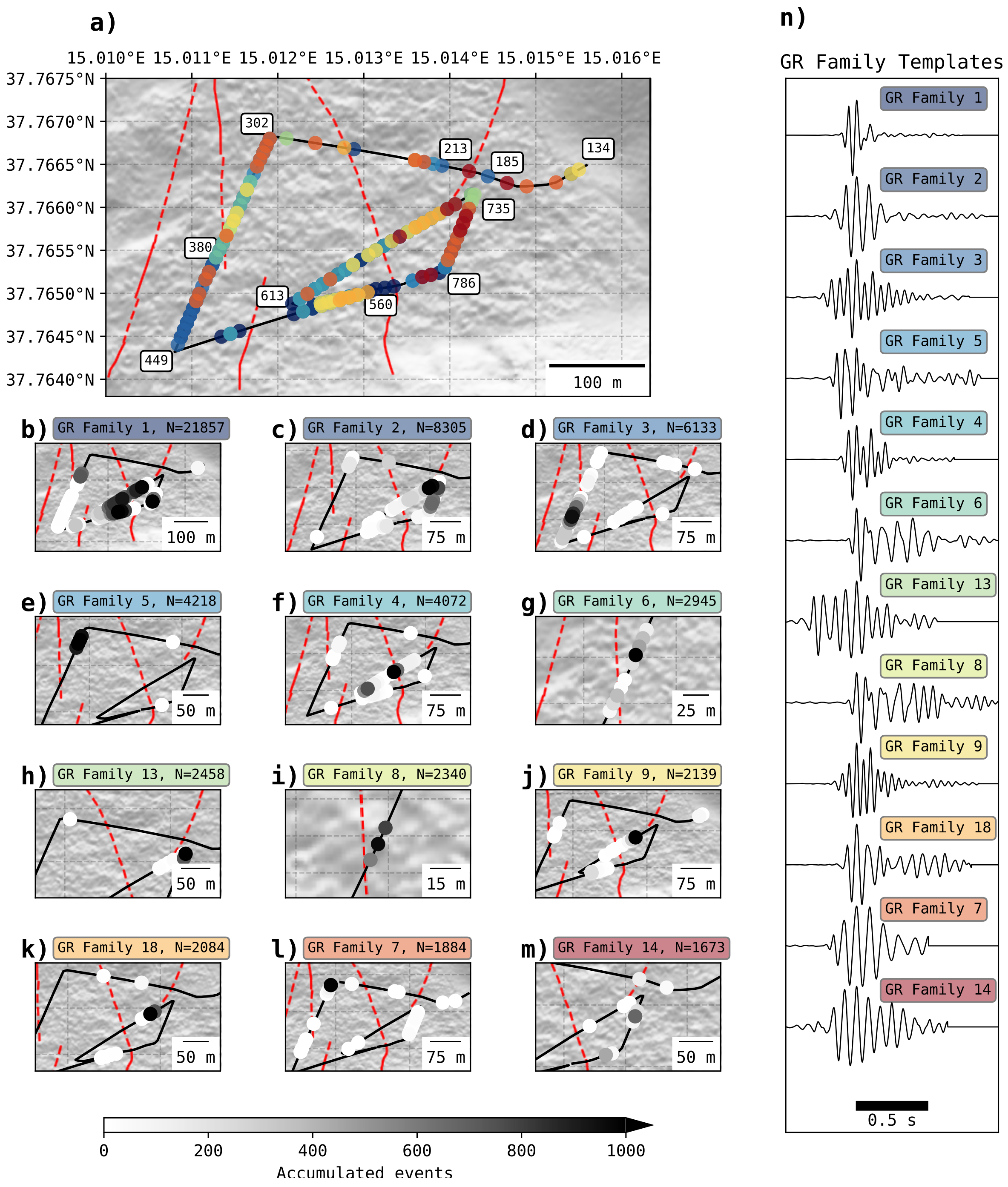

Figure S11: Spatial distribution of the first 12 GR families with the highest number of members. GR families were obtained using a spatial classification with a lower CC threshold (0.75) than the one in the main manuscript (0.87). **a)** Overview map illustrating the presence of the first 12 GR families along the fibre optic cable (black curve) with distinctive colours for each GR family. Colours are connected to the labels of each GR family in subfigures **b - m)** and **n)**. **b - m)** Zoomed view showing the local distribution of each GR family and their occurrence (number of accumulated events) at each channel where they are present, indicated by colour scale (lower part). **n)** Obtained waveform templates (normalized amplitude) from GR classification for each of the 12 GR families.

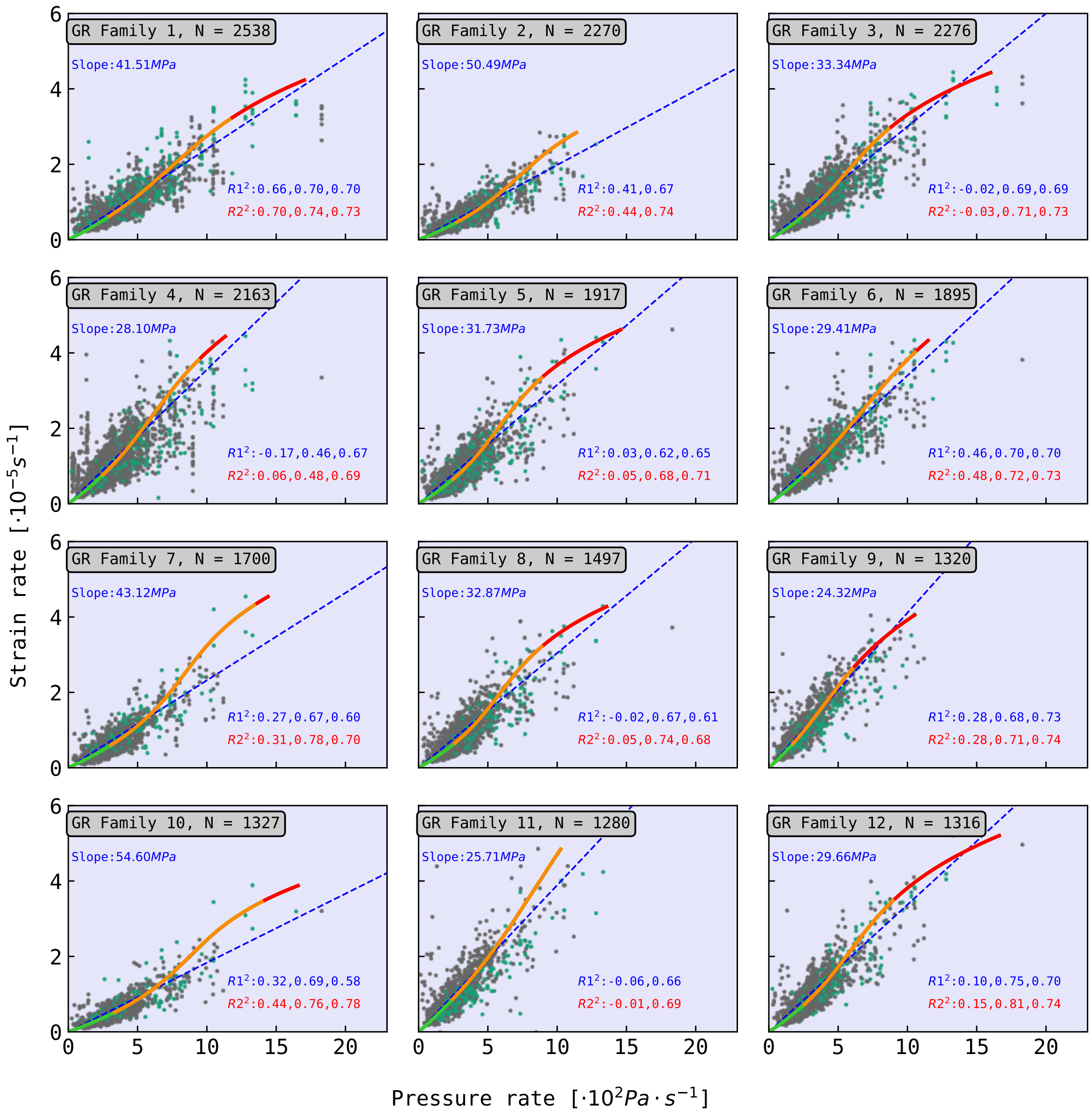

Figure S12: Strain rate of GR events with all channels associated to the GR families with the highest number of members vs. pressure rate of volcanic explosions from explosion family 1. Color of dots represents explosions and consequent ground response observed during day (green; 06:00 to 18:00 CET) and night (green; 18:00 to 06:00 CET). Each plot presents a linear fitting curve (dashed blue line) and a 3rd order nonlinear curve (green-orange-red curve). The nonlinear curve represents the hyperleastic interpretation. The hyperplastic curve is composed by three stages: initial elastic behaviour (Stage 1: green curve), softening process (Stage 2: orange curve), and posterior stiffening (Stage 3; red curve). Each fitting curve has its R^2 score reported: R1 for linear fitting, and R2 for nonlinear. for each elastic stage, there is an individual R^2 score reported in order of the elastic stages.

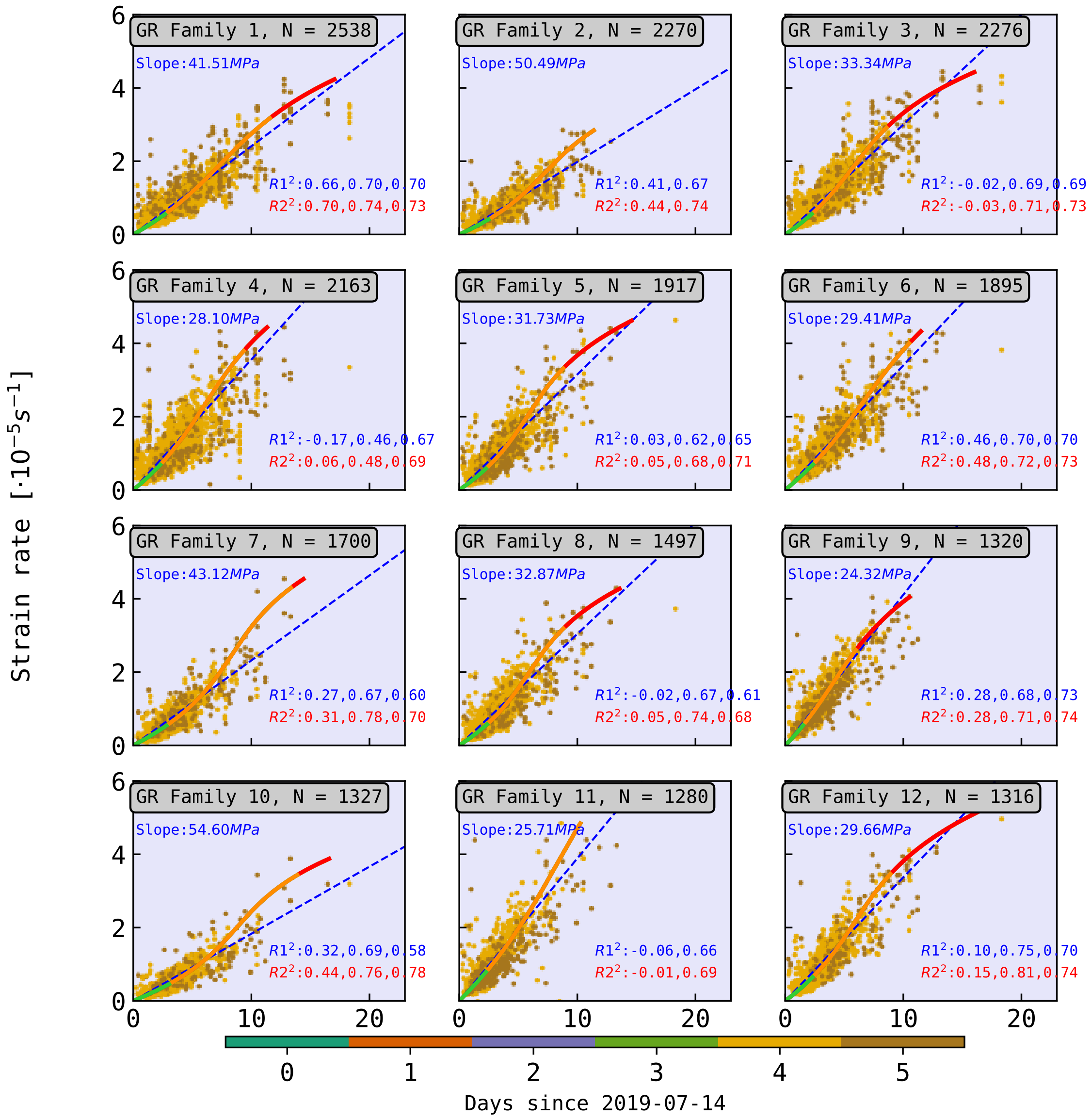

Figure S13: Peak-to-peak (p-p) strain rate of DDSS ground response events vs. p-p pressure rate of the high frequency components (10-50 Hz) from volcanic explosion (infrasound) events related to family 1. Color of dots represents the number of the day after 14th July of 2019. The first column shows the strain rate vs. pressure rate relation for all infrasound events (black dots) of family 1 at four distinctive channels (rows). The rest of the columns show the biggest ground response family for the respective channel (blue dots) over the ground response residual events (grey dots).

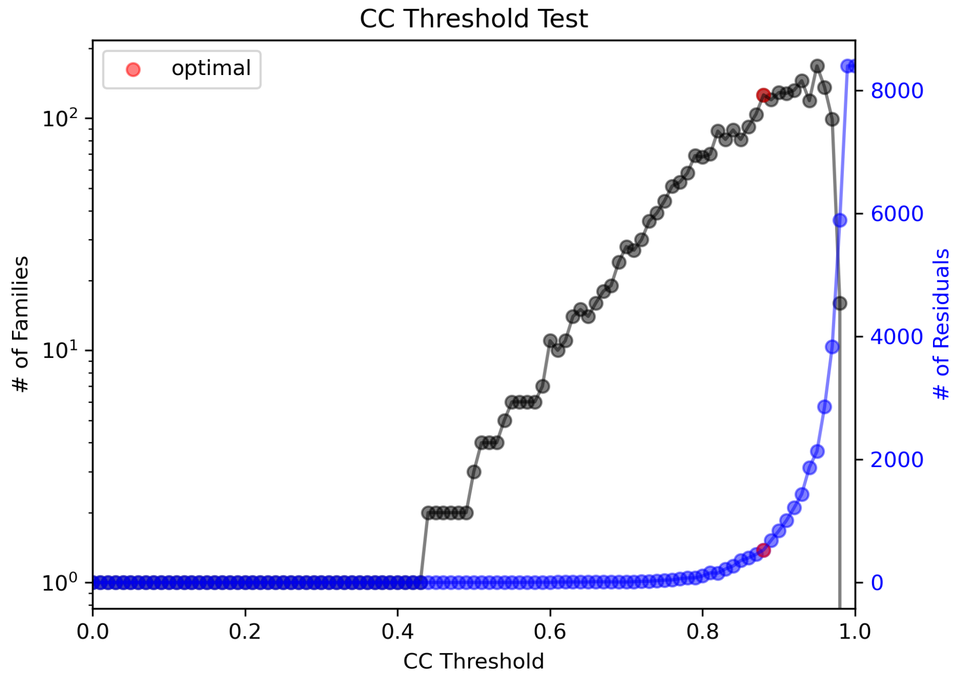

Figure S14: Number of explosion families achieved and residuals (non-classified explosion waveforms) for different cross-correlation (CC) thresholds across for explosions in infrasound (sensor ARB1). Red dot indicate the inflection points found by automatic elbow method (CC threshold of 0.88).

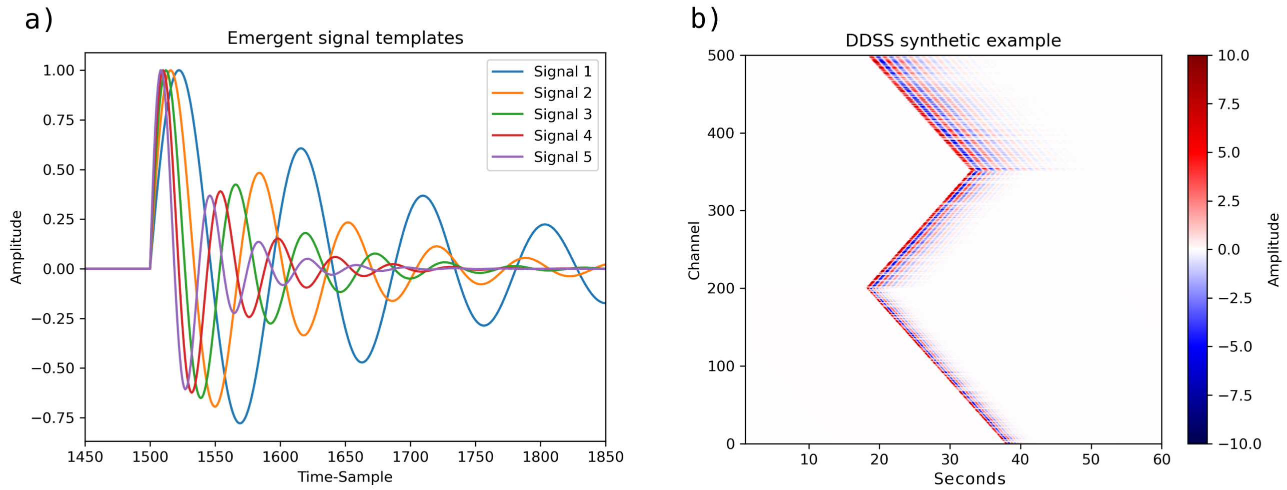

Figure S15: Synthetic signals created for validating the GR classification method. A total of 5 templates were created **(a)**. The templates were used to create synthetic DDSS events **(b)**. Signal templates **(a)** are used with modulated amplitude to simulate coupling and GR signal strength. A moveout is introduced between ranges of channels (0-200, 200-360 and 360-500) to mimic change in cable directionality. A total of 60 synthetic DDSS events were made to test the classification method (see Methods: Ground response classification in main text.)

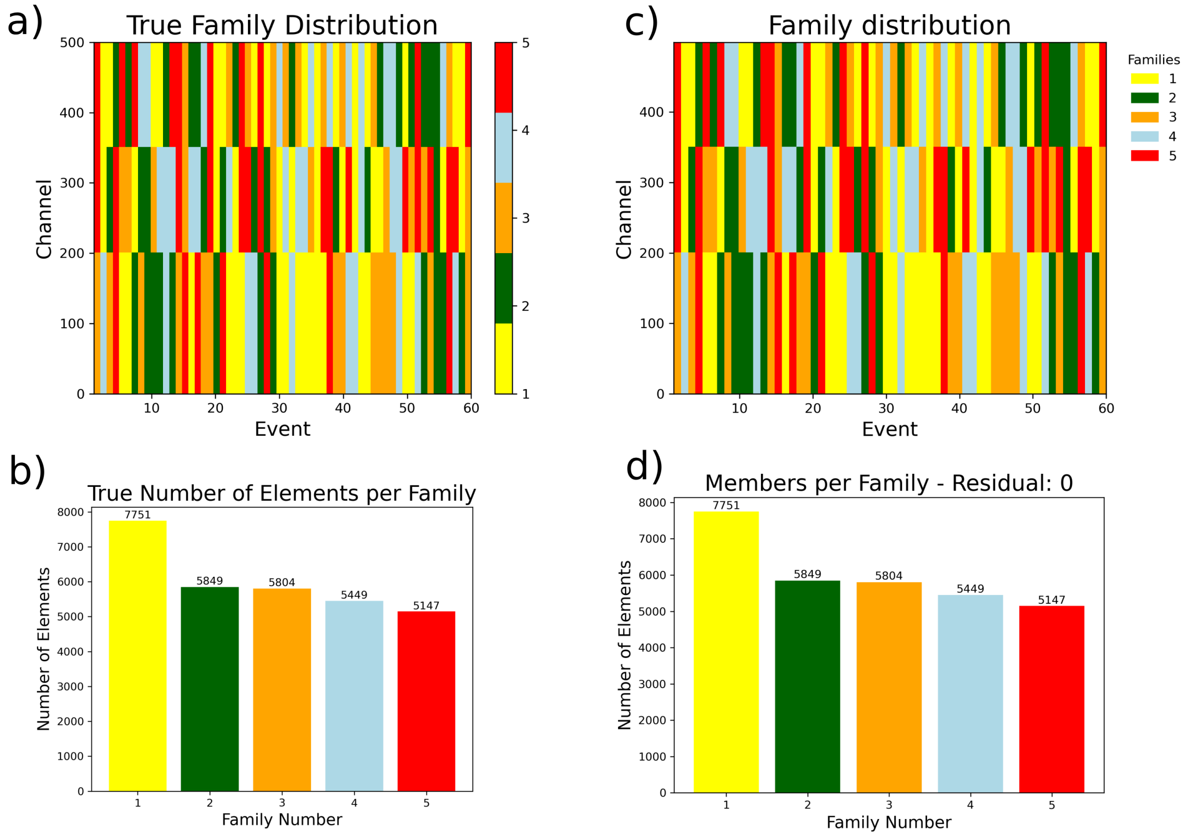

Figure S16: Validation of DDSS classification using synthetics S15. **a)** and **b)** are the true distribution of families across channels, and their respective number of members per family, respectively. **c)** and **d)** are the obtained distribution of families across channels with the modified DDSS classification method, and the number of members per family, respectively. The method demonstrates its capability to retrieve the solution of DDSS waveform distributions present in DDSS data.

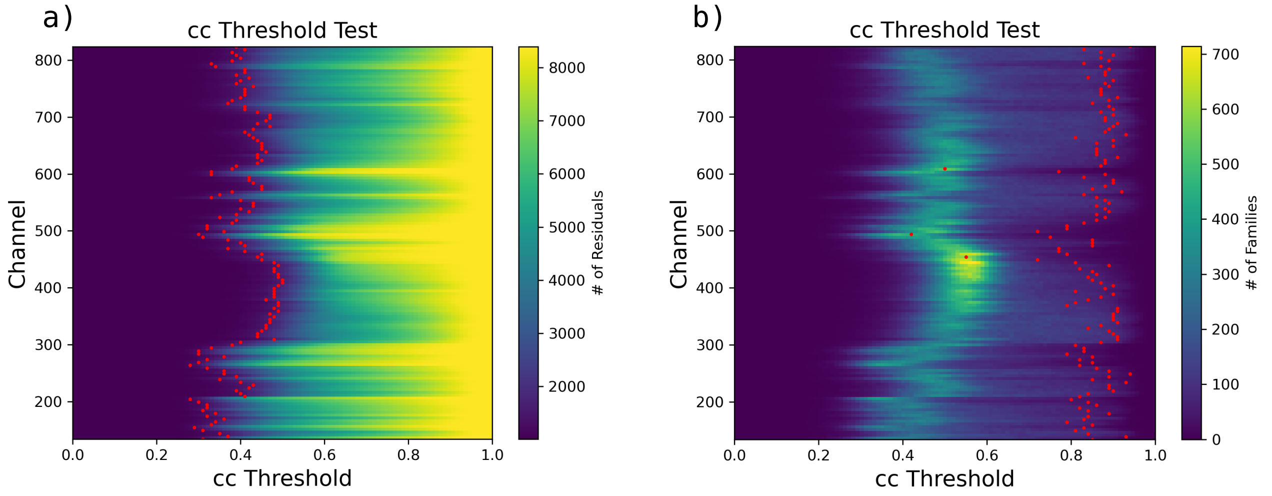

Figure S17: Classification test at each channel. **a)** Number of residuals (non-classified GR waveforms) achieved for different cross-correlation (CC) thresholds for step 5 (Fig. 8 of main text) for GR data in DDSS. Red dot indicate the inflection point found by automatic elbow method. **b)** Number of GR families achieved for different cross-correlation (CC) thresholds for step 5 (Fig. 8 of main text) for GR data in DDSS. Red dots indicate local maxima while maximizing CC threshold value.

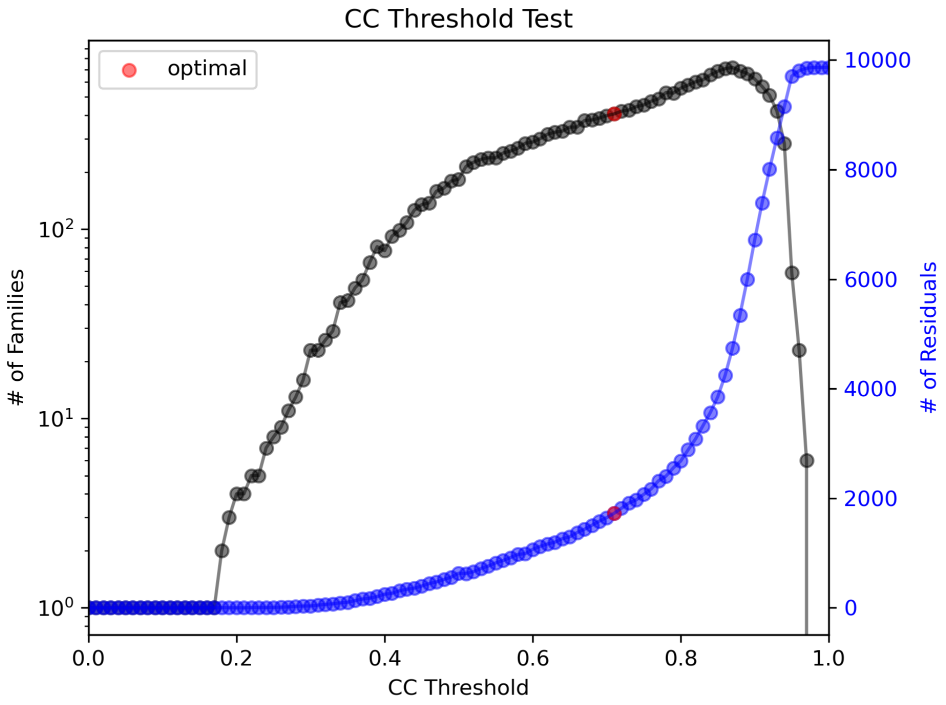

Figure S18: Number of GR families achieved and residuals (non-classified GR waveforms) for different cross-correlation (CC) thresholds for step 5 (Fig. 9 of main text) for GR data in DDSS. Red dot indicate the inflection point found by automatic elbow method (CC 0.71). The performance at 0.71 CC was no convincing after an inspection of the resulted GR templates in comparison to its members. Therefore, the CC threshold chosen for classification was for the score achieving the maximum number of families (CC 0.87).

2 Supplementary Tables

**Variable**	**iDAS (standard fiber)**
Sampling Frequency[Hz]	1000.0
Spatial Resolution[m]	2.0
Gauge Length	10.0
Start Position[m]	119.774209
Measure Length[m]	1664
Stream Time[s]	inf
Continuous Mode	True
Source Mode	Normal
Power Decrement	0.0
Start Distance (m)	-124.020952
Stop Distance (m)	1574.843176
Precise Sampling Frequency (Hz)	20000.0
Peak Voltage[V]	2.0
Pulse 2 Delay (ns)	500.0
Pulse Width [ns]	50.0
OffsetLength	64.0
PreTrig Samples	94

Reference length	0
Saving Bandwidth (MB/s)	350.0
Reference Level 1	65535
Reference Level 2	49030
Reference Level 3	50530
Fibre Index	1.4682
Fibre Length Multiplier	1.020952
Unit Calibration (nm)	116.0
Diff Amplify Factor	0
Attenuator 2	2.31502
Fibre Length per Metre	1.0
Zero Offset (m)	-124.02095
Receiver Gain	66.00 66.00 66.00
Pulse Width 2 (ns)	50.0
Peak Voltage 2 (V)	0.0
Attenuator 1	0.0
Time Decimation	20
Output Decimation [bool]	4
P	5
P Coefficients	1.0 x 5
Integration Cut Off (Hz)	3.14
Normalization	True
Decimation Filter	True
Acoustic Output	Differential
Window	0
Defragmented	False
iDAS Version	2.4.1.102

Table S1: Metadata and acquisition parameters for the survey done with the iDAS unit. No change was made since acquisition was continuous.
